# Early Progression of Abdominal Aortic Aneurysm is Decelerated by Improved Endothelial Barrier Function via ALDH2‐LIN28B‐ELK3 Signaling

**DOI:** 10.1002/advs.202302231

**Published:** 2023-10-11

**Authors:** Kehui Yang, Sumei Cui, Jingwen Wang, Tonghui Xu, Han Du, Hongwei Yue, Huaqing Ye, Jialin Guo, Jian Zhang, Pengpai Li, Yunyun Guo, Chang Pan, Jiaojiao Pang, Jiali Wang, Xiao Yu, Cheng Zhang, Zhiping Liu, Yuguo Chen, Feng Xu

**Affiliations:** ^1^ Department of Emergency Medicine Chest Pain Center Qilu Hospital of Shandong University Jinan Shandong 250012 China; ^2^ Shandong Provincial Clinical Research Center for Emergency and Critical Care Medicine Key Laboratory of Emergency and Critical Care Medicine of Shandong Province Key Laboratory of Cardiopulmonary‐Cerebral Resuscitation Research of Shandong Province Shandong Provincial Engineering Laboratory for Emergency and Critical Care Medicine Qilu Hospital of Shandong University Jinan Shandong 250012 China; ^3^ The Key Laboratory of Cardiovascular Remodeling and Function Research Chinese Ministry of Education Chinese Ministry of Health and Chinese Academy of Medical Sciences The State and Shandong Province Joint Key Laboratory of Translational Cardiovascular Medicine Qilu Hospital of Shandong University Jinan Shandong 250012 China; ^4^ Department of Biomedical Engineering School of Control Science and Engineering Shandong University Jinan Shandong 250012 China; ^5^ Key Laboratory Experimental Teratology of the Ministry of Education and Department of Physiology and Pathophysiology School of Basic Medical Sciences Shandong University Jinan Shandong 250012 China; ^6^ Department of Cardiology Qilu Hospital of Shandong University Jinan Shandong 250012 China

**Keywords:** abdominal aortic aneurysm, aldehyde dehydrogenase 2, ELK3, endothelial cells, initiation

## Abstract

The involvement of endothelial barrier function in abdominal aortic aneurysm (AAA) and its upstream regulators remains unknown. Single‐cell RNA sequencing shows that disrupted endothelial focal junction is an early (3 days) and persistent (28 days) event during Angiotensin II (Ang II)‐induced AAA progression. Consistently, mRNA sequencing on human aortic dissection tissues confirmed downregulated expression of endothelial barrier‐related genes. Aldehyde dehydrogenase 2 (ALDH2), a negative regulator of AAA, is found to be upregulated in the intimal media of AAA samples, leading to testing its role in early‐stage AAA. ALDH2 knockdown/knockout specifically in endothelial cells (ECs) significantly increases expression of EC barrier markers related to focal adhesion and tight junction, restores endothelial barrier integrity, and suppresses early aortic dilation of AAA (7 and 14 days post‐Ang II). Mechanically, ELK3 acts as an ALDH2 downstream regulator for endothelial barrier function preservation. At the molecular level, ALDH2 directly binds to LIN28B, a regulator of ELK3 mRNA stability, hindering LIN28B binding to ELK3 mRNA, thereby depressing ELK3 expression and impairing endothelial barrier function. Therefore, preserving vascular endothelial barrier integrity via ALDH2‐specific knockdown in ECs holds therapeutic potential in the early management of AAAs.

## Graphical abstract

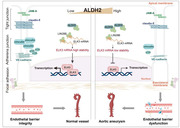

## 1 Introduction

Abdominal aortic aneurysm (AAA) is an abnormal bulge in the wall of the largest artery that carries blood from the heart to the body. AAA is fatal in over 60% of cases without surgical intervention,^[^ ^1^, ^2^, ^3^ ^]^ is often being discovered incidentally through imaging or screening programs,^[^ ^4^, ^5^, ^6^, ^7^ ^]^ and current management strategies are limited to lifestyle modifications, medication, and surveillance.^[^ ^3^, ^8^, ^9^ ^]^ Surgery is typically recommended when the diameter of the aneurysm exceeds 5.5 cm or when it is >4.5 cm for patients with bicuspid aortic valve stenosis or regurgitation.^[^ ^10^ ^]^ However, over 60% of patients have aneurysms that are <5.5 cm, yet ≈40% of these aneurysms may still rupture unexpectedly.^[^ ^3^, ^5^, ^11^, ^12^, ^13^, ^14^ ^]^ Investigating its underlying pathogenesis is therefore important to inform development of therapeutic targets for aneurysmal formation and deceleration of early aortic aneurysm progression, thereby preventing its rupture and minimizing the need for surgery.

AAA is characterized by loss of vascular smooth muscle cells (VSMCs), extracellular matrix destruction, and intraluminal thrombosis.^[^ ^15^, ^16^, ^17^ ^]^ Ample therapeutic targets have been identified based on the aforementioned pathological features.^[^ ^18^, ^19^ ^]^ Nonetheless, a contribution from endothelial cells (ECs) has received little attention despite its potential importance in early risk management of aortic aneurysms.^[^ ^17^ ^]^ The vascular endothelium plays a crucial role as the primary defense mechanism against hemodynamic and hormonal stimuli.^[^ ^20^, ^21^ ^]^ Its central function is to act as a regulated barrier, primarily controlled by intercellular junctions, including tight junctions and adherens junctions, as well as extracellular attachments like focal adhesions.^[^ ^22^ ^]^ Tight junctions consist of several proteins including claudins, junctional adhesion molecules (JAMs), and occludin. Adherens junctions, on the other hand, are protein complexes that are composed of transmembrane proteins such as vascular endothelial‐cadherin (VE‐cadherin), vinculin, and p120‐catenin. Focal adhesions, which are special points of attachment between the endothelial basolateral membranes and the extracellular matrix, are composed of focal adhesion kinase 1 (FAK) and focal adhesion kinase 2 (FAK2). To date, many studies have documented critical causal involvement of endothelial barrier function in the progression of thoracic aortic aneurysm and dissection (TAAD) with disrupted endothelial barrier function facilitating the infiltration of inflammatory cells and accumulation of excess fluid in aortic layers.^[^ ^23^, ^24^, ^25^, ^26^ ^]^ However, little attention has been given to the endothelial barrier during the formation of AAA.^[^ ^27^ ^]^ Since aortic anatomical location plays a critical role in the pathogeneses of aortic aneurysm and dissection (AAD) and mouse models of AAD have limited gene profile overlap, we aimed to investigate the participation of endothelial barrier function in abdominal aortas using an Angiotensin II (Ang II)‐induced AAA animal model.^[^ ^28^ ^]^

Aldehyde dehydrogenase 2 (ALDH2), which is traditionally regarded as a mitochondrial enzyme, plays a critical role in a variety of cardiovascular pathologies mainly through detoxifying endogenous and exogenous aldehydes.^[^ ^29^ ^]^ In addition, recent studies provided new insights into the non‐enzymatic roles of ALDH2 in cardiovascular diseases.^[^ ^2^, ^30^, ^31^ ^]^ ALDH2 can translocate from mitochondria to the nucleus, to regulate genes important for cholesterol biosynthesis in hepatocytes, and efferocytosis in macrophages.^[^ ^30^, ^31^, ^32^ ^]^ We previously identified that ALDH2 deficiency significantly reduced the incidence of AAD partly by preventing vascular smooth muscle cell phenotype switch.^[^ ^2^ ^]^ However, little information is available on the possible influence of ALDH2 on endothelial barrier and early AAA expansion. Herein, we aimed to decipher the effect of endothelial barrier defect during early‐stage AAA and whether it is subject to ALDH2 regulation to pinpoint potential modulators of endothelial barrier function as therapeutic targets for early‐stage AAA.

## 2 Results

### 2.1 Endothelial Barrier Dysfunction is an Early and Long‐Lasting Pathological Event in AAA Development

**Figure 1:**
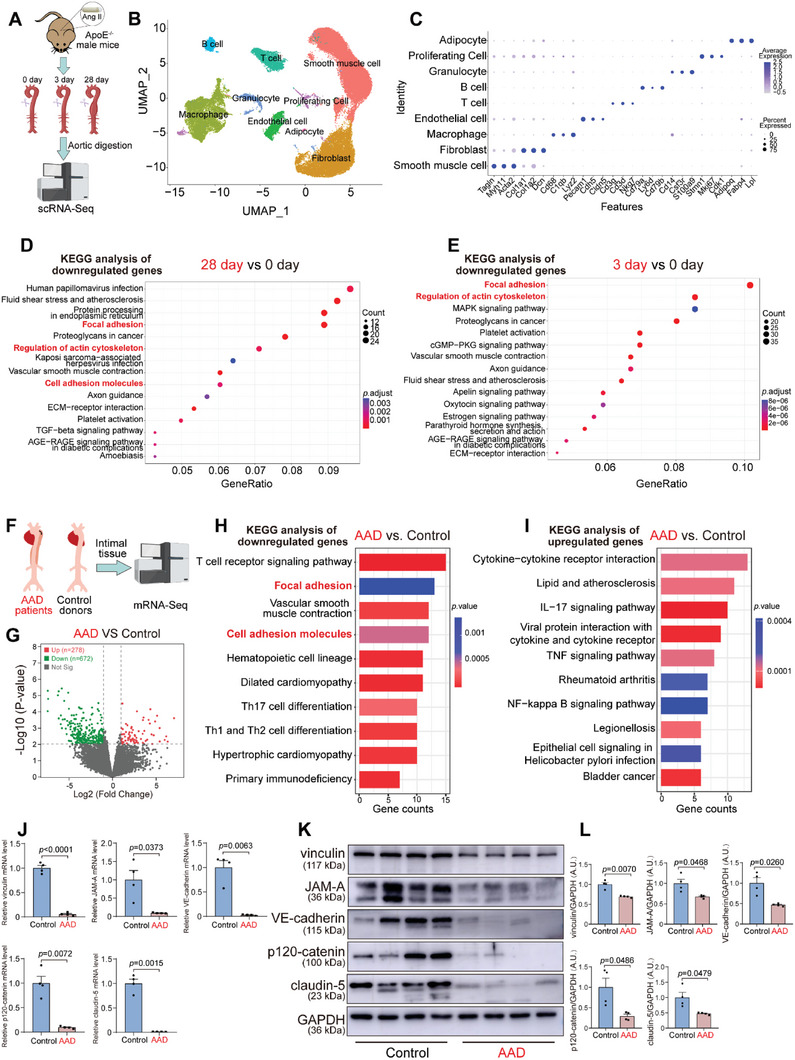
Endothelial barrier pathway is an early and long‐lasting pathological event in the development of abdominal aortic aneurysm. A) Experimental strategy of single cell RNA‐sequencing (scRNA‐seq) to derive a dynamic transcriptome of aortic cells. Male ApoE^−/−^ mice were categorized into day 0 (*n* = 5 aortas), day 3 (*n* = 5 aortas), and day 28 groups (*n* = 5 aortas) based on the varying durations of Angiotensin II (Ang II) exposure. Full‐length aortas were digested to obtain single‐cell suspensions for sequencing with DNBelab C Series High‐throughput Single‐cell System (BGI‐research). B) Uniform Manifold Approximation and Projection (UMAP) plot identified nine distinct cell populations of aortas from day 0 without Ang II exposure, or 3, 28 days after Ang II exposure (day 0, day 3, and day 28 combined). C) A dot plot analysis indicating the relative expression of marker genes in distinct cell populations. Dot size reflects the percentage of cells expressing the selected gene in each population and dot color corresponds to expression level. D) A list of top 15 downregulated pathways from KEGG enrichment analysis in endothelial cells on day 28 versus day 0. E) A list of the top 15 downregulated pathways from KEGG enrichment analysis in endothelial cells on day 3 versus day 0. F) Schematic of mRNA‐seq to derive a detailed transcriptome of aortic intimal tissue isolated from human aortic aneurysm/dissection (AAD) patients and control donors. G) Volcano plot exhibiting differentially expressed genes in intimal tissues between AAD and control individuals. H) A list of top ten downregulated pathways from KEGG enrichment analysis in AAD aortic intimal tissues versus control aortic intimal tissues. I) A list of top ten upregulated pathways from KEGG enrichment analysis in AAD aortic intimal tissues versus control aortic intimal tissues. J) RT‐qPCR of vinculin, JAM‐A, VE‐cadherin, p120‐catenin, and claudin‐5 (*n* = 4 per group). K,L) Representative immunoblots and quantification for vinculin, JAM‐A, VE‐cadherin, p120‐catenin, and claudin‐5 (*n* = 4 per group). Data are presented as mean ± SEM. Unpaired two‐tailed Student's t‐test was used in vinculin of (J); unpaired two‐tailed Student's t‐tests with Welch's correction were used in the rest markers of (J,L). A.U. indicates arbitrary unit.

Due to the difficulty in acquiring early‐stage human AAA tissues and aortic cellular heterogeneity, single cell RNA‐sequencing (scRNA‐seq) was utilized to derive endothelial cell‐specific dynamic transcriptional patterns across AAA development trajectories in Ang II‐induced AAA models (**Figure** **1**A). Aortas were harvested at day 0 without Ang II exposure, as well as 3 and 28 days after Ang II exposure. Following enzymatic digestion, aortic cell suspensions with stringent quality control were sequenced using a DNBelab C Series High‐throughput Single‐cell System (BGI‐research, Figure S1A,B, Supporting Information). Unsupervised Seurat‐based clustering identified 9 distinct cell populations from Ang II‐infused aortas at different time points (Figure 1B). All aortic cells were defined by 9 cell clusters shown by the dot plot and the top markers were identified based on genome alignment with CellRanger involving classic marker genes, such as Tagln, Myh11 and Acta2 for smooth muscle cells; Col1a1, Col1a2, and Dcn for fibroblasts; Cd68, C1qb, Lyz2 for macrophages; and Pecam 1, Cdh5 and Cldn5 for endothelial cells, Cd3g, Cd3d for T cells; Cd79a, Ly6d, Cd79b for B cells; Cd14, Csf3r, and S100a9 for granulocytes; Stmn1, Mki67, and Cdk1 for proliferating cells; and Adipoq, Fabp4 and Lpl for adipocyte cells (Figure 1C). We then directed our focus toward ECs and analyzed differentially downregulated genes. Kyoto Encyclopedia of Genes and Genomes (KEGG) pathway analysis revealed notable alterations in focal adhesion, regulation of actin cytoskeleton, and cell adhesion molecules pathway at both days 3 and 28 after Ang II infusion (Figure 1D,E); focal adhesion pathway and regulation of actin cytoskeleton were the two most significantly enriched functional clusters on day 3 as evidenced by the elevated Gene ratio score (Figure 1E). Analysis of gene ontology terms related to biological processes revealed that cell‐substrate adhesion, regulation of cell‐substrate adhesion, cell‐matrix adhesion, and cell junction assembly were among the top downregulated pathways at days 3 and 28 after Ang II infusion (Figure S1C,D, Supporting Information). Moreover, in western blot analysis the levels of vinculin, JAM‐A, VE‐cadherin, p120‐catenin, and claudin‐5 gradually decreased at serial time points after Ang II infusion (Figure S1E,F, Supporting Information). These results suggested that pathways contributing to ECs barrier function were significantly downregulated, and that early and long‐lasting EC barrier dysfunction was involved in mouse AAA development.

In human AAD intimal tissues, mRNA sequencing identified 278 upregulated genes and 672 downregulated genes compared with control intimal tissues (Figure 1F,G). Based on the differentially downregulated genes, KEGG pathway analysis revealed that focal adhesion and cell adhesion molecule pathways were significantly altered (Figure 1H). Consistent with previous studies, KEGG pathway analysis of differentially upregulated genes revealed that cytokine–cytokine receptor interaction pathway and lipid and atherosclerosis pathway were upregulated (Figure 1I). RT‐qPCR and western blot experiments further confirmed lower levels of the barrier mediators vinculin, JAM‐A, VE‐cadherin, p120‐catenin, and claudin‐5 in human AAD intimal tissues (Figure 1J–L). These results confirmed the involvement of endothelial barrier dysfunction in aortic aneurysmal progression. Altogether, a dysregulated endothelial barrier pathway was an early and long‐lasting pathological event during AAA progression.

### 2.2 ALDH2 is Upregulated in Intima of Aortic Aneurysms

**Figure 2:**
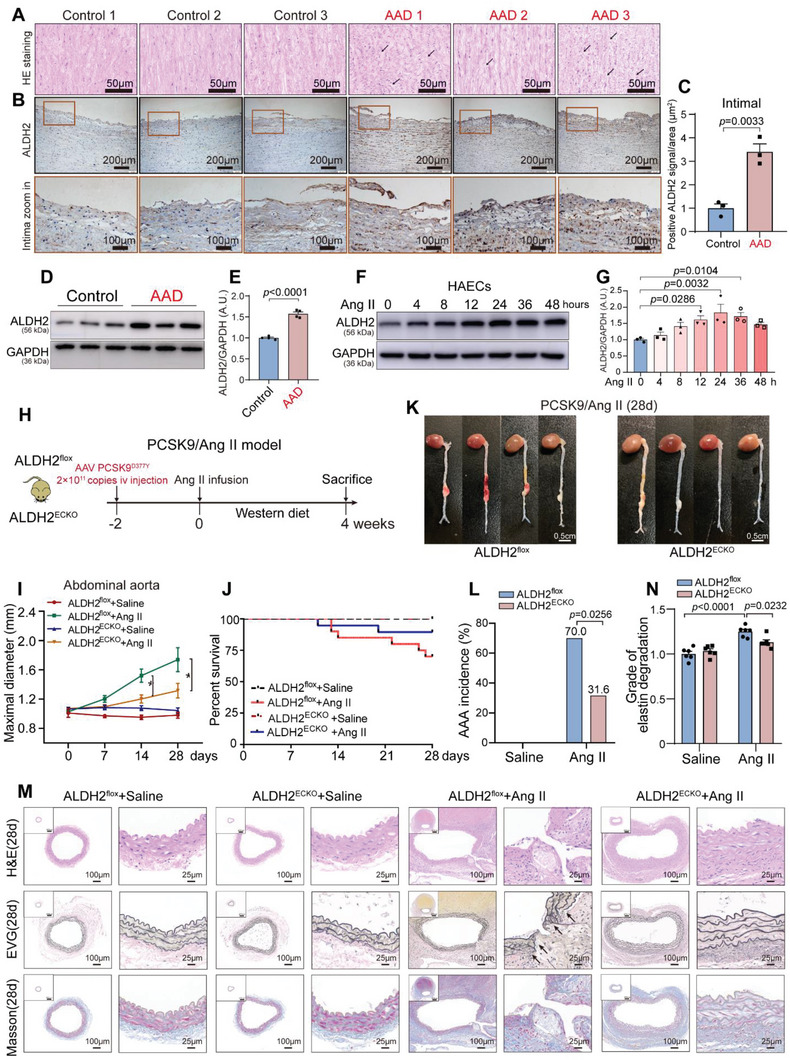
ALDH2 is increased in the human AAD intima and ECs‐specific ALDH2 knockout suppresses aortic dilation in the early stage of Ang II‐induced AAA. A) Representative hematoxylin and eosin (H&E) staining in the aortic tissues from AAD patients (*n* = 3) and controls (*n* = 3). The black arrows refer to degraded elastic fibers. Scale bar, 50 µm. B) Representative ALDH2 immunohistochemical staining images in the intima of aortic tissues from AAD patients (*n* = 3) and controls (*n* = 3). Scale bar of low‐magnification images, 200 µm. Scale bar of intima zoom in images, 100 µm. C) Quantitative analysis of ALDH2 positive staining signals on panel B (*n* = 3 per group). D,E) Representative immunoblots and quantification of ALDH2 in aortic intima tissues from AAD patients and controls (*n* = 4 per group). F,G) Representative immunoblots and quantification of ALDH2 in Ang II‐treated human aortic endothelial cells (HAECs, *n* = 3 per group). (H) Schematic protocol: AAV8‐PCSK9^D377Y^ was injected prior to infusion of Ang II or saline in mice. I) Maximal aortic diameter in four male animal groups. Day 0: ALDH2^flox^ + Saline, n = 6; ALDH2^ECKO^ + Saline, *n* = 7; ALDH2^flox^ + Ang II, n = 20; and ALDH2^ECKO^ + Ang II, *n* = 19. Day 7: ALDH2^flox^ + Saline, *n* = 6; ALDH2^ECKO^ + Saline, *n* = 7; ALDH2^flox^ + Ang II, *n* = 17; and ALDH2^ECKO^ + Ang II, *n* = 18. Day 14: ALDH2^flox^ + Saline, *n* = 6; ALDH2^ECKO^ + Saline, *n* = 7; ALDH2^flox^ + Ang II, *n* = 15; and ALDH2^ECKO^ + Ang II, *n* = 18. Day 28: ALDH2^flox^ + Saline, *n* = 5; ALDH2^ECKO^ + Saline, *n* = 7; ALDH2^flox^ + Ang II, *n* = 11; and ALDH2^ECKO^ + Ang II, *n* = 16. ALDH2^flox^ versus ALDH2^ECKO^ group 14 days post‐Ang II treatment, *p = 0.0009; ALDH2^flox^ versus ALDH2^ECKO^ group 28 days post‐Ang II treatment, *p < 0.0001. J) Survival curves in indicated groups. ALDH2^flox^ + Saline, *n* = 6; ALDH2^ECKO^ + Saline, *n* = 7; ALDH2^flox^ + Ang II, *n* = 20; and ALDH2^ECKO^ + Ang II, *n* = 19. K) Representative macroscopic images between ALDH2^flox^ and ALDH2^ECKO^ mice 28 days post‐Ang II treatment. Scale bar, 0.5 cm. L) AAA incidence in four male animal groups 28 days post‐Ang II treatment. ALDH2^flox^ + Saline, *n* = 6; ALDH2^ECKO^ + Saline, *n* = 7; ALDH2^flox^ + Ang II, *n* = 20; and ALDH2^ECKO^ + Ang II, *n* = 19. M) Representative H&E, EVG staining and Masson staining of mouse abdominal aorta in indicated groups 28 days post‐Ang II treatment. The black arrows refer to degraded elastic fibers. Low‐magnification images in M show the entire vascular wall at the site of analysis. Scale bar of high‐magnification images in M, 100 and 25 µm. N) Grade of elastin degradation in the aortic wall. Data are presented as mean ± SEM. Unpaired two‐tailed Student's t tests were used in (C,E). One‐way ANOVA followed by Bonferroni post hoc test was applied in (G). Two‐way ANOVA followed by Bonferroni post hoc analysis was applied in (I,N). Fisher's Exact Test was applied in (L). A.U. indicates arbitrary unit.

Earlier findings from our group noted that ALDH2 inhibition overtly reduces AAD incidence in part by preventing SMC phenotype switch.^[^ ^2^ ^]^ Here, ALDH2 expression was scrutinized in the pathogenesis and progression of AAA. We verified that human AAD specimens were histologically characterized by degraded elastic fibers (**Figure** **2**A). Of note, ALDH2 was upregulated in the human AAD intima by immunohistochemistry staining (Figure 2B,C). Consistently, immunoblot assays showed that ALDH2 protein levels were dramatically increased in human AAD intimal tissues compared with controls (Figure 2D,E). ALDH2 levels also progressively increased in Ang II‐challenged human aortic endothelial cells (HAECs) (Figure 2F,G). Although ALDH2 was upregulated in the intima of AAD tissues, a direct link between the expression of ALDH2 and the endothelial barrier remains to be established.

### 2.3 EC‐Specific ALDH2 Knockout/Knockdown Suppresses Aortic Dilation at an Early Stage and Ultimately Prevents AAA Formation

EC‐specific ALDH2 knockout mice (ALDH2^ECKO^) mice with a confirmed genotype of ALDH2^flox^Tie2‐Cre and ALDH2^flox^ were utilized as experimental groups, and ALDH2^flox^ mice were used as the control group (Figure S2, Supporting Information). The mice were administered an adeno‐associated virus (AAV8) vector to overexpress Proprotein Convertase Subtilisin/Kexin Type 9 (PCSK9^D377Y^), a gain‐of‐function mutation. The mice were then exposed to a high‐fat diet and subjected to Ang II infusion to induce AAA^[^ ^33^ ^]^ (Figure 2H). ECs‐specific ALDH2 knockout did not alter the aortic diameter and histological morphology prior to Ang II treatment (Figure S3, Supporting Information), and did not change metabolic parameters 4 weeks after Ang II challenge (Figure S4A–E, Supporting Information). Of note, ALDH2^ECKO^ mice showed significantly suppressed aortic dilation at days 7, 14, and 28 post AAA induction (Figure 2I; Figure S4F, Supporting Information). Moreover, ALDH2^ECKO^ mice showed a significantly increased cumulative survival rate (Figure 2J) and markedly reduced AAA incidence (31.6%), compared with ALDH2^flox^ mice (AAA incidence of 70.0%; Figure 2K,L). H&E and EVG staining indicated that EC‐specific ALDH2 knockout reduced elastin disruption and degradation in the aortic wall at days 28 post AAA induction (Figure 2M,N). Masson staining also showed that the degree of collagen deposition induced by Ang II was reduced by EC‐specific ALDH2 knockout (Figure 2M).

AAV is promising in gene therapy for its stable, efficient, and non‐cytotoxic gene delivery and is one of the most commonly used viral vectors in gene therapy.^[^ ^34^ ^]^ We adopted AAV1‐Recombinant Intercellular Adhesion Molecule2 (ICAM2)‐shALDH2 system to selectively knockdown ALDH2 in ECs on ApoE^−/−^ mice (Figure S5A, Supporting Information). AAA was established by high fat feeding plus Ang II infusion (Figure S5B, Supporting Information), which is considered to recapitulate the major features of human AAA.^[^ ^35^, ^36^ ^]^ Strong GFP fluorescence signals in ECs confirmed the successful viral delivery 4 weeks after AAV1‐ICAM2 injection into ApoE^−/−^ mice (Figure S5C, Supporting Information). Significant knockdown of ALDH2 was confirmed by immunofluorescence and western blot in mouse ECs following administration of AAV1‐ICAM2 carrying shALDH2 compared with those carrying AAV1‐scramble (Figure S5D,E, Supporting Information). In this system, EC‐specific ALDH2 knockdown did not affect metabolic parameters 4 weeks after Ang II challenge (Figure S5F–J, Supporting Information). Of special note, AAV1‐shALDH2 significantly suppressed aortic dilation at days 7, 14, and 28 post AAA induction (Figure S5K, Supporting Information). At the end of experiment, AAV1‐shALDH2 administration resulted in an increased cumulative survival rate (Figure S5L, Supporting Information) and reduced AAA incidence (30.0%) more dramatically compared with AAV1‐scramble (AAA incidence of 63.6%) after Ang II infusion (Figure S5M,N, Supporting Information). H&E, EVG staining, and Masson staining evinced that EC‐specific ALDH2 knockdown inhibited Ang II induced pathological changes in abdominal aorta (Figure S5O,P, Supporting Information). These data suggest that ECs‐specific ALDH2 knockdown suppressed aortic dilation in early‐stage AAA and ultimately prevents AAA formation.

### 2.4 EC‐Specific ALDH2 Knockout/Knockdown Protects Endothelial Barrier Function In Vivo and In Vitro

**Figure 3:**
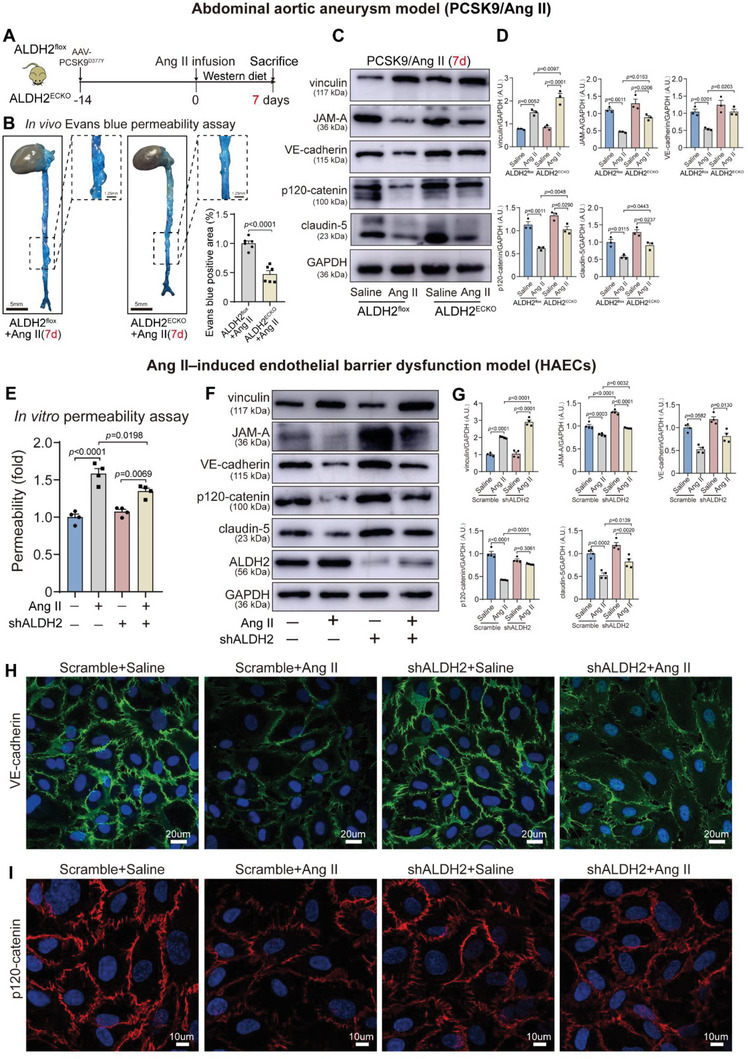
EC‐specific ALDH2 knockdown/knockout alleviates Ang II‐induced endothelial barrier dysfunction in vivo and in vitro. A) Schematic protocol: AAV8‐PCSK9^D377Y^ was injected prior to infusion of Ang II or saline in mice. B) Permeability of aortic intimal barrier (to Evans blue dye) and quantification of the Evans blue dye in ALDH2^flox^ and ALDH2^ECKO^ mice 7 days post‐Ang II treatment. ALDH2^flox^ + Ang II, *n* = 6; and ALDH2^ECKO^ + Ang II, *n* = 6. Scale bars, 5 mm. C,D) Representative immunoblots and quantification for vinculin, JAM‐A, VE‐cadherin, p120‐catenin, and claudin‐5 in the indicated groups (*n* = 3 per group). E) In vitro permeability assay for HAECs monolayers (shALDH2 or scramble) after 24 h of Ang II (10^−6^ m) treatment (*n* = 4 per group). F,G) Representative immunoblots and quantification for vinculin, JAM‐A, VE‐cadherin, p120‐catenin, claudin‐5, and ALDH2 in indicated groups (*n* = 4 per group). H,I) VE‐cadherin‐specific antibody staining (green, scale bars, 20 µm) and I) p120‐catenin‐specific antibody staining (red, scale bars, 10 µm) of Ang II–transfected HAECs. Data are presented as mean ± SEM. Unpaired two‐tailed Student's t tests were used in (B). Two‐way ANOVA followed by Bonferroni post hoc analyses were applied in (D,E,G). A.U. indicates arbitrary unit.

To investigate the role of ALDH2 in maintaining endothelial barrier function, we compared endothelial barrier function in the early stage of AAA between ALDH2^ECKO^ and ALDH2^flox^ mice (**Figure** **3**A). Using in vivo Evans blue permeability assay, ALDH2^ECKO^ mice showed less appearance of Evans blue dye in the aorta compared with ALDH2^flox^ mice 7 days after Ang II treatment (Figure 3B). Vinculin is a cytoplasmic actin‐binding protein enriched in focal adhesions and adherens junctions that is upregulated to repair the impaired EC barrier.^[^ ^37^ ^]^ Western blot on protein extracts from these aortic tissues demonstrated that Ang II infusion provoked a remarkable higher level of vinculin, the effect of which was also greatly improved by ECs‐specific ALDH2 knockout (Figure 3C,D). Furthermore, ECs‐specific ALDH2 knockout restored downregulation of the barrier mediators including JAM‐A, VE‐cadherin, p120‐catenin, and claudin‐5 in the face of Ang II challenge on day 7 post Ang II treatment (Figure 3C,D).

A murine endothelial injury model was established by Ang II infusion for 2 weeks in ALDH2^−/−^ mice with C57BL/6J genetic background (Figure S6A, Supporting Information). ALDH2^−/−^ mice showed less appearance of Evans blue dye in the aorta (Figure S6B, Supporting Information). Western blot analysis revealed that ALDH2 deletion was associated with an even higher level of vinculin provoked by Ang II infusion and restored downregulation of the barrier mediators including JAM‐A, VE‐cadherin, p120‐catenin, and claudin‐5 in the face of Ang II challenge (Figure S6C,D, Supporting Information). In line with western blot results, immunofluorescence data suggested an upregulated claudin‐5 expression in response to Ang II in ALDH2^−/−^ aortic tissues (Figure S6E, Supporting Information).

Using in vitro experiments, primary HAECs were treated with various concentrations of Ang II for different durations. Twenty‐four hours of 10^−6^ m Ang II was chosen as optimal timing to dramatically downregulate barrier mediator expression (Figure S7, Supporting Information). An in vitro permeability assay was included to examine the function of ALDH2 on endothelial permeability. As shown in Figure 3E, Ang II treatment increased HAEC permeability, although the effect was largely attenuated by shALDH2 silencing (Figure 3E). Western blot analysis revealed that shALDH2 was associated with an even higher level of vinculin after Ang II infusion and reversed Ang II‐induced down‐regulation of JAM‐A, VE‐cadherin, p120‐catenin, and claudin‐5 (Figure 3F,G). Moreover, in analysis of other adhesion molecules involved in EC adhesion, shALDH2 reduced levels of phosphorylated β‐catenin and paxillin (Figure S8, Supporting Information). Consistently, immunofluorescence assessment further revealed reduced expression of barrier mediators, VE‐cadherin (green) and p120‐catenin (red) by Ang II, which returned to near normal levels with shALDH2 maneuver (Figure 3H,I). In line with shALDH2 data, the ALDH2 inhibitor Daidzin greatly attenuated endothelial permeability (Figure S9A,B, Supporting Information) and reversed down‐regulation of endothelial barrier mediators after Ang II treatment (Figure S9C–F, Supporting Information). In summary, downregulated ALDH2 is protective of endothelial barrier function in vitro.

### 2.5 Downregulated ALDH2 Increases ELK3 Expression to Preserve Endothelial Barrier Function

**Figure 4:**
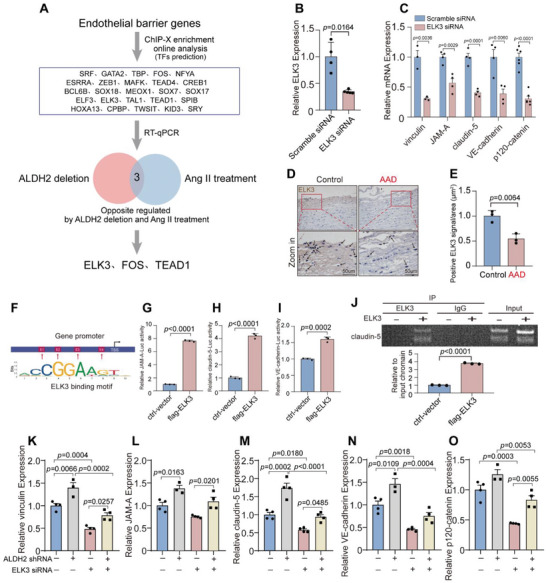
Downregulated ALDH2 increases ELK3 expression to preserve endothelial barrier function. A) The ChIP‐X Enrichment Analysis 3 (ChEA3) online tool was used to predict the top 25 MeanRank transcription factors (TFs) as potential EC barrier regulators. These 25 TFs were experimentally verified in aortic tissue and HUVECs, among that ELK3, FOS, and TEAD1 were verified as oppositely regulated by ALDH2 deletion and Ang II treatment. B) RT‐qPCR of ELK3 in HUVECs transfected with negative control siRNA (scramble siRNA) or ELK3‐siRNA (*n* = 4 per group). C) RT‐qPCR of vinculin, JAM‐A, claudin‐5, VE‐cadherin, and p120‐catenin in HUVECs transfected with scramble siRNA or ELK3‐siRNA (*n* = 3–6 per group). D,E) Representative immunostaining and quantification for ELK3 in the intima from AAD patients and controls (*n* = 3 per group). The black arrows refer to positive ELK3 signal. Scale bar of zoom in images, 50 µm. F) Scheme of predicted ELK3 binding sites in human JAM‐A, claudin‐5, and VE‐cadherin promoter. TSS: transcription start site. G) Luciferase assay exhibiting interplay between ELK3 and JAM‐A promoter (*n* = 3 per group). H) Luciferase assay exhibiting interplay between ELK3 and claudin‐5 promoter (*n* = 3 per group). I) Luciferase assay exhibiting interplay between ELK3 and VE‐cadherin promoter (*n* = 3 per group). J) Chromatin immunoprecipitation assay of ELK3 binding to claudin‐5 gene promoters in HUVECs with or without ELK3 overexpression by plasmid transfection (*n* = 3 per group). K) RT–qPCR of vinculin, L) JAM‐A, M) claudin‐5, N) VE‐cadherin, and O) p120‐catenin in HUVECs treated with shALDH2 or ELK3‐siRNA after Ang II treatment (*n* = 4 per group). Data are presented as mean ± SEM. Unpaired two‐tailed Student's t tests with Welch's correction was used in (B) and unpaired two‐tailed Student's t tests were used in (C,E,G–J). Two‐way ANOVA followed by Bonferroni post hoc analysis was applied in (K–O).

We went on to explore the molecular mechanism of ALDH2 in endothelial barrier regulation. The ChIP‐X Enrichment Analysis 3 (ChEA3) tool (https://amp.pharm.mssm.edu/ChEA3) was used to predict the top 25 MeanRank transcription factors (TFs) as potential EC barrier regulators (**Figure** **4**A).^[^ ^38^ ^]^ Among them, RT‐qPCR analysis chose FOS, ELK3, and TEAD1 as potential mediators, which were as oppositely regulated by ALDH2 deletion (Figure S10A, Supporting Information) and Ang II treatment (Figure S10B, Supporting Information). Compared with the other two TFs, ELK3 siRNA displayed the most significant and consistent effects on downregulating endothelial barrier genes in vitro (Figure 4B,C; Figure S10C–I, Supporting Information). Moreover, human AAD aortic intimal tissues exhibited downregulated ELK3 protein level by immunostaining assessment (Figure 4D,E). Studies demonstrated that ELK3 functions as a transcriptional repressor that, when phosphorylated, is transformed into a transcriptional activator.^[^ ^39^, ^40^ ^]^ We questioned whether phosphorylated ELK3 might affect the expressions of endothelial barrier genes. As shown in Figure S10J,K (Supporting Information), shALDH2 reversed Ang II‐induced down‐regulation of phosphorylated ELK3 and total ELK3 in HUVECs. Based on these findings, we hypothesized that ELK3 may function as a transcriptional activator by some unknown mechanism and was chosen for further analysis. Importantly, using bioinformatical analysis, ELK3 was confirmed to be vital in cell–cell junction, actin cytoskeleton, focal adhesion, cell‐substrate adherens junction, cell‐substrate, and adherens junction in the MDA‐MB‐231 cell line (Figure S11, Supporting Information). Luciferase‐based reporter assay revealed that ELK3 increased the transcriptional activity of JAM‐A, claudin‐5, and VE‐cadherin promoters (Figure 4F–I). Furthermore, overexpression of ELK3 increased the binding between ELK3 and claudin‐5 promoter in ChIP assay (Figure 4J). These data suggested a seemingly vital role for ELK3 for endothelial barrier and was thus chosen for further mechanistic experiments.

Using Ang II‐treated HUVECs as in vitro model, shALDH2 or siELK3 were transfected to further investigate the role of ELK3 in ALDH2 mediating endothelial barrier function. In Ang II‐treated HUVECs, shALDH2 restored the impairment of endothelial barrier markers vinculin (Figure 4K), JAM‐A (Figure 4L), claudin‐5 (Figure 4 M), VE‐cadherin (Figure 4N), and p120‐catenin (Figure 4O), and the latter effects were abolished by ELK3 siRNA (Figure 4K–O). These in vitro data suggest that ELK3 is required in the barrier functional improvement mediated by ALDH2 downregulation.

### 2.6 ELK3 Acts as ALDH2 Downstream Target to Inhibit Aortic Aneurysm Progression at Early Stages

**Figure 5:**
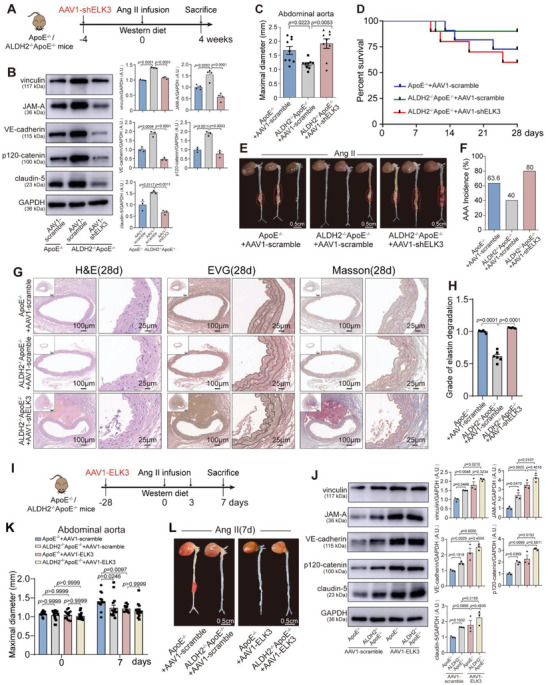
ELK3 serves as an ALDH2 downstream target to inhibit aortic aneurysm progression at early stages. A) Schematic protocol displaying injection of AAV1‐shELK3 or AAV1‐scramble into ApoE^−/−^ mice and ALDH2^−/−^ApoE^−/−^ mice prior to Ang II or saline infusion. B) Representative immunoblots and quantification for vinculin, JAM‐A, VE‐cadherin, p120‐catenin, and claudin‐5 in the indicated groups (*n* = 3–4 per group). C) Maximal aortic diameter 4 weeks post‐Ang II treatment. ApoE^−/−^+AAV1‐scramble, *n* = 9; ALDH2^−/−^ApoE^−/−^+AAV1‐scramble, *n* = 9; and ALDH2^−/−^ApoE^−/−^+AAV1‐shELK3, *n* = 9. D) Survival curves in indicated groups. ApoE^−/−^+AAV1‐scramble, *n* = 11; ALDH2^−/−^ApoE^−/−^+AAV1‐scramble, *n* = 10; and ALDH2^−/−^ApoE^−/−^+AAV1‐shELK3, *n* = 10. E) Representative macroscopic images in indicated groups after Ang II treatment for 4 weeks. Scale bar, 0.5 cm. F) AAA incidence 4 weeks post‐Ang II treatment. ApoE^−/−^+AAV1‐scramble, *n* = 11; ALDH2^−/−^ApoE^−/−^+AAV1‐scramble, *n* = 10; and ALDH2^−/−^ApoE^−/−^+AAV1‐shELK3, *n* = 10. G) Representative H&E, EVG staining and Masson staining of the mouse aortas in indicated groups 4 weeks post‐Ang II treatment. Low‐magnification images in G show the entire vascular wall at the site of analysis. Scale bar of high‐magnification images in G, 100 and 25 µm. H) Grade of elastin degradation in the aortic wall. I) Schematic protocol exhibiting injection of AAV1‐ELK3 or AAV1‐scramble into ApoE^−/−^ mice and ALDH2^−/−^ApoE^−/−^ mice prior to Ang II or saline infusion. J) Representative immunoblots for vinculin, JAM‐A, VE‐cadherin, p120‐catenin, and claudin‐5 in the indicated groups (*n* = 3–4 per group). K) Maximal aortic diameter at 7 days post‐Ang II treatment. ApoE^−/−^+AAV1‐scramble, *n* = 15; ALDH2^−/−^ApoE^−/−^+AAV1‐scramble, *n* = 15. ApoE^−/−^+AAV1‐ELK3, *n* = 15; ALDH2^−/−^ApoE^−/−^+AAV1‐ELK3, *n* = 15. L) Representative macroscopic images in indicated groups 7 days post‐Ang II treatment. Scale bar, 0.5 cm. Data are presented as mean ± SEM. One‐way ANOVA followed by Bonferroni post hoc test was applied in (B) and one‐way ANOVA followed by Brown‐Forsythe and Welch ANOVA test was applied in (C,H). Two‐way ANOVA followed by Bonferroni post hoc analysis was applied in (K) (J). A.U. indicates arbitrary unit.

We next investigated whether the effect of ALDH2 knockout on maintenance of endothelial barrier function and AAA development was ELK3 dependent. EC‐specific ELK3 knockdown was achieved in vivo using AAV1‐ICAM2‐shELK3 (Figure S12A, Supporting Information), and animal studies were performed following the protocol shown in **Figure** **5**A. Significant inhibition of ELK3 expression in mouse ECs was confirmed by immunofluorescence 4 weeks after AAV1‐ ICAM2 carrying shELK3 injection into ApoE^−/−^ mice (Figure S12B, Supporting Information). Western blot analysis revealed that ALDH2 knockout markedly augmented levels of ELK3, vinculin, JAM‐A, VE‐cadherin, p120‐catenin, and claudin‐5, whereas EC‐specific ELK3 knockdown diminished the expression of ELK3 and these endothelial barrier markers (Figure S12C, Supporting Information; Figure 5B). We found that ALDH2 knockout eliminated AngII‐induced abdominal aortic diameter at days 28 post AAA induction compared with ApoE^−/−^ mice, whereas EC‐specific ELK3 knockdown significantly increased abdominal aortic dilation (Figure 5C; Figure S12D, Supporting Information). Moreover, EC‐specific ELK3 knockdown showed a significantly decreased cumulative survival rate (Figure 5D) and increased AAA incidence (Figure 5E,F) compared with mice injected with AAV1‐scramble. H&E, EVG staining, and Masson staining noted that EC‐specific ELK3 knockdown augmented the degradation of elastic fibers and the degree of collagen deposition in abdominal aorta (Figure 5G,H). Thus, ELK3 is necessary to maintain endothelial barrier function in AAA.

We examined the role of ELK3 in regulating EC barrier function and early AAA. To manipulate ELK3, ApoE^−/−^ and ALDH2^−/−^ApoE^−/−^ mice were preincubated with AAV1‐ELK3 for 4 weeks to selectively overexpress ELK3 in ECs before Ang II exposure (Figure S13A–C, Supporting Information; Figure 5I). We first compared endothelial barrier function in the early stages of AAA between ApoE^−/−^ and ALDH2^−/−^ApoE^−/−^ mice. Western blot analysis confirmed that ELK3 overexpression abolished the ALDH2 effect on the reduction of endothelial barrier markers vinculin, JAM‐A, claudin‐5, VE‐cadherin, and p120‐catenin at day 7 post‐Ang II treatment (Figure 5J). It is noteworthy that pretreatment with AAV1‐ELK3 abolished the accelerated effect of ALDH2 in aortic dilation (Figure 5K,L; Figure S13D, Supporting Information), degradation of elastic fibers and the degree of collagen deposition (Figure S13E,F, Supporting Information) after Ang II infusion, indicating ELK3 acted as the ALDH2 downstream to restrict endothelial dysfunction in early progression of AAA. Hence, we conclude that ELK3 is required in the barrier functional improvement mediated by ALDH2 downregulation.

### 2.7 ALDH2 Binds with LIN28B to Decrease ELK3 mRNA Stability

**Figure 6:**
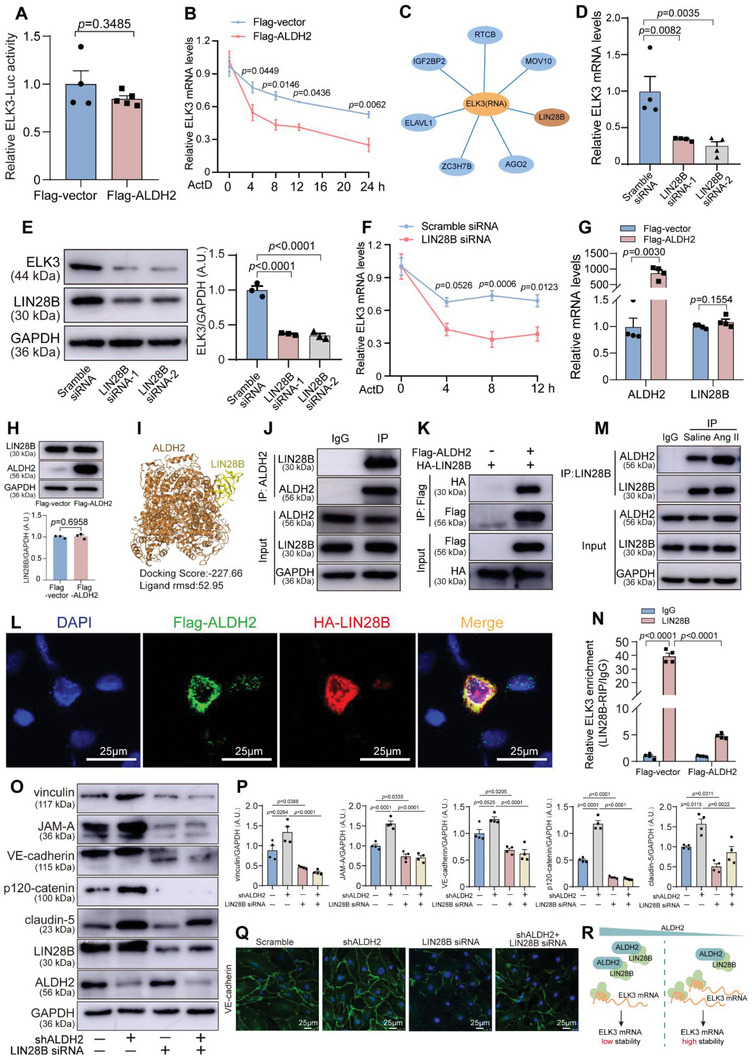
Downregulated ALDH2 upregulates ELK3 expression through LIN28B‐mediated mRNA stability. A) Luciferase assay exhibiting relationship between ALDH2 and ELK3 promoter (*n* = 4 per group). B) The ELK3 mRNA decay in HUVECs was evaluated using RT‐qPCR following pretreatment with actinomycin D to inhibit transcription (*n* = 6 per group). C) Potential proteins mediating the ELK3 mRNA stability by the RNA‐protein interaction database. D) RT‐qPCR of ELK3 in HUVECs treated with LIN28B siRNA (n = 4 per group). E) Representative immunoblots and quantification for LIN28B and ELK3 in LIN28B siRNA‐treated HUVECs (*n* = 3 per group). F) RT‐qPCR was utilized to determine ELK3 mRNA decay in HUVECs after pretreatment with actinomycin D to inhibit transcription (*n* = 4 per group). G) RT‐qPCR of ALDH2 and LIN28B in HUVECs treated with a flag‐ALDH2 plasmid (*n* = 4 per group). H) Representative immunoblots and quantification for ALDH2 and LIN28B in flag‐ALDH2 plasmid‐transfected HUVECs (*n* = 3 per group). I) Visualization of ALDH2‐ LIN28B interacting interface prediction based on the Hdock tool. Cartoon representation depicts the complex structures with the predicted interaction residues. J) Co‐immunoprecipitation assay of ALDH2 and LIN28B in HUVECs (*n* = 3 per group). K) Flag‐ALDH2 was co‐transfected with HA‐LIN28B into HEK293T. Cell lysates were subjected to Co‐IP with an anti‐Flag antibody, followed by western blotting (*n* = 3 per group). L) Representative confocal microscopic images of colocalization of ALDH2 and LIN28B in Hela cells. Scale bar, 25 µm. M) Co‐immunoprecipitation assay of ALDH2 and LIN28B in HUVECs cell line treated with Ang II (*n* = 3 per group). N) RNA immunoprecipitation assay was performed using HUVECs cell lysates. RT‐qPCR was performed to measure relative LIN28B binding to ELK3 mRNA using primers designed for ELK3. Samples immunoprecipitated with immunoglobulin G (IgG) served as control (*n* = 4 per group). O,P) Representative immunoblots and quantification for vinculin, JAM‐A, VE‐cadherin, p120‐catenin, claudin‐5, LIN28B, and ALDH2 in HUVECs treated with shALDH2 or LIN28B siRNA after 24 h Ang II treatment (*n* = 4 per group). Q) VE‐cadherin‐specific antibody staining (green) in HUVECs treated with shALDH2 or LIN28B siRNA after 24 h Ang II treatment (*n* = 3 per group). Scale bar, 25 µm. R) Schematic diagram of the regulation mechanism of ELK3 mRNA stability. Data are presented as mean ± SEM. Unpaired two‐tailed Student's t tests with Welch's correction were used in (A,G) and unpaired two‐tailed Student's t test was used in (H). Two‐way ANOVA followed by Bonferroni post hoc analyses were applied in (B,F,N,P). One‐way ANOVA followed by Bonferroni post hoc tests were applied in (D,E). A.U. indicates arbitrary unit.

ALDH2 was previously reported to translocate from mitochondria to nucleus, regulating downstream genes at the transcriptional level.^[^ ^30^ ^]^ However, immunofluorescence experiments showed that Ang II treatment or ALDH2 inhibition by Daidzin failed to alter nuclear translocation of ALDH2 (Figure S14A,B, Supporting Information), thereby excluding the possibility of ALDH2 translocation in our system. At the transcriptional level, the luciferase reporter assay demonstrated that ALDH2 overexpression did not change the transcriptional activity of the ELK3 promoter (**Figure** **6**A). Next, we measured mRNA stability after transcriptional inhibition with Actinomycin D treatment and surprisingly found that ALDH2 negatively regulated ELK3 mRNA stability (Figure 6B).

As little evidence was available regarding direct contribution of ALDH2 to mRNA stability, we assumed an indirect interaction via an RNA binding protein existed between them. The RNA‐protein interaction database (http://lulab.life.tsinghua.edu.cn/postar2/index.php) predicted seven RNA binding proteins that could potentially mediate the interaction between ALDH2 and ELK3 mRNA (Figure 6C).^[^ ^41^ ^]^ Among these RBPs, Lin‐28 Homolog B (LIN28B) had the highest score in the entire genomic context, utilizing both its cold shock and zinc finger RNA binding domains to preferentially interact with various mRNAs, thereby regulating gene expression at the post‐transcriptional level.^[^ ^42^, ^43^, ^44^ ^]^ To verify protein function of LIN28B in our system, we performed high‐resolution mass spectrometry‐based proteomics that revealed a catalog of proteins that are bind with LIN28B. Based on the 178 differentially expressed proteins, KEGG pathway analysis noted that these proteins were mainly involved in mRNA metabolism and modification pathways (Figure S14C, Supporting Information). The chemical reactions and pathways involving metabolism of mRNA, mRNA metabolism process, translation factors, deadenylation of mRNA, which is responsible for carrying the coded genetic “message” to sites of protein assembly at the ribosomes to enhance a function for which the coded protein is required (Figure S14C, Supporting Information). Moreover, Co‐IP analysis revealed that LIN28B interacts with RNA helicase A (RHA) thereby modulating the translation of target mRNAs via interactions with eukaryotic translation initiation factors (eIFS) and elongation factors (eFFS), among others (Figure S14D, Supporting Information). These data showed that LIN28B is responsible for the regulation of gene expression. Further studies were aimed at exploring whether LIN28B was required for ELK3 function.

Our data revealed that LIN28B siRNA repressed ELK3 expression at both the mRNA (Figure 6D; Figure S14E, Supporting Information) and protein levels (Figure 6E). After pretreatment with actinomycin D to suppress transcription, we noted that LIN28B siRNA decreased the stability of ELK3 mRNA (Figure 6F). ALDH2 overexpression did not alter LIN28B expression (Figure 6G,H), neither did Ang II treatment (Figure S14F, Supporting Information). We hypothesized that elevated ALDH2 protein bound more LIN28B, thereby antagonizing the binding between ELK3 mRNA and LIN28B under Ang II infusion. We explored a potential regulatory effect of ALDH2 on LIN28B. The cartoon representation of the complex structures with the predicted interaction interface according to the Hdock tool are illustrated in Figure 6I. Co‐immunoprecipitation (Co‐IP) assay showed that endogenous ALDH2 and LIN28B were physically associated in HUVECs (Figure 6J). HEK293T cells were transfected with ALDH2 and LIN28B plasmids, respectively, and in Co‐IP analysis, ALDH2 co‐precipitated with LIN28B (Figure 6K). Immunofluorescence experiments showed that ALDH2 was able to bind to LIN28B directly (Figure 6L). Most importantly, Ang II treatment fostered the binding between ALDH2 and LIN28B (Figure 6M) and ALDH2 inhibition by Daidzin treatment decreased the binding (Figure S14G, Supporting Information). ALDH2 overexpression antagonized the binding between ELK3 mRNA and LIN28B (Figure 6N). Overall, the latter observations indicate that binding between LIN28B and ELK3 mRNA is negatively regulated by ALDH2.

To discern possible involvement of ALDH2‐LIN28B interaction in regulating endothelial barrier function, we assessed levels of endothelial barrier markers in HAECs with ALDH2 knockdown accompanied by introduction of LIN28B siRNA into cells. Western blot and immunofluorescence analyses revealed that LIN28B siRNA abolished upregulation of ALDH2 knockdown‐induced endothelial barrier markers (Figure 6O–Q). Collectively, these data demonstrate that ALDH2 negatively regulates the binding between LIN28B and ELK3 mRNA that destabilizes the latter thereby impairing the endothelial barrier (Figure 6R).

## 3 Discussion

**Figure 7:**
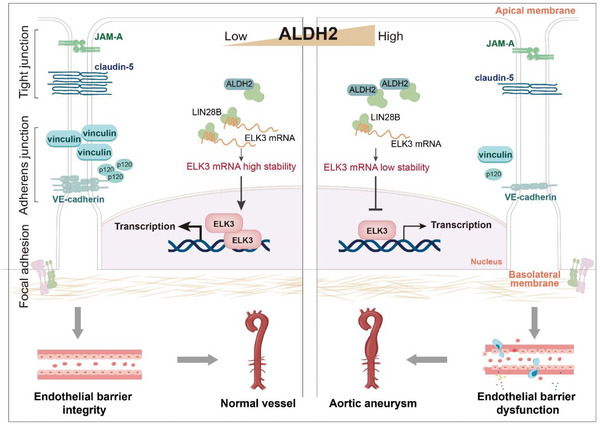
ALDH2 upregulation is detrimental to vascular endothelial barrier integrity and promotes the early progression of AAA. Under pathological conditions, ALDH2 expression level is elevated, competitively decreasing the binding between LIN28B and ELK3 mRNA, thereby destabilizing ELK3 mRNA and inhibiting endothelial barrier gene expression, which promotes endothelial hyperpermeability and accelerates the development of early AAA.

Our study has identified, for the first time, a causal link between endothelial barrier function in the abdominal regions and Ang II‐induced AAA. More importantly, our present research evinced that downregulated ALDH2 in ECs is a novel therapeutic method for preserving endothelial barrier function in early AAA progression. Mechanistically, ALDH2 directly binds with LIN28B, a crucial mediator of RNA stability, thereby hindering LIN28B from binding to the mRNA for ELK3, which results in decreased activity as a key transcriptional factor of multiple EC barrier markers (as shown in **Figure** **7**). This finding has significant implications for developing new therapeutic strategies for treating AAA, a life‐threatening and heterogeneous disease with different etiologies.

To date, many studies have linked endothelial barrier dysfunction to TAA.ECs depleted for SMAD6 present with dysregulated junction related genes and defective barrier function in TAA development.^[^ ^45^ ^]^ Either ROBO4 (a cell–cell adhesion mediator) silencing or mutant ROBO4 expression in ECs also resulted in impaired barrier function and the pathogenesis of TAA.^[^ ^46^ ^]^ Furthermore, loss of ADAM17 in ECs protected the integrity of the intimal barrier consequently preventing TAA progression.^[^ ^47^ ^]^ S‐nitrosylation modification of the endothelial PLS3 protein was identified in TAA(D) tissues, and in vitro studies showed that this modification contributes to vascular endothelial barrier dysfunction.^[^ ^26^ ^]^ The above studies indicated that endothelial barrier dysfunction is closely related to TAA. However, the cause‐effect relationship between endothelial barrier function and AAA remains elusive until recently, when a study reported that the disruption of endothelial tight junction is an early event prior to TAA(D) formation and revealed the critical causal involvement of endothelial barrier function in the progression of TAA(D).^[^ ^23^ ^]^ It is important to note that various mouse models reflect different aspects of the human aortic aneurysm/dissection pathology.^[^ ^28^ ^]^ For instance, BAPN mainly induces thoracic aortic dissection, while Ang II and CaPO_4_ promotes the development of AAA.^[^ ^48^, ^49^ ^]^ The limited overlap between the gene profiles found in the CaCl_2_ and Ang II models also highlights the idea that different models might reflect different aortic pathologies.^[^ ^28^ ^]^ Therefore, it is crucial to explore the participation and upstream regulators in other models of AAA before developing therapeutic methods that target the disruption of endothelial barrier function. Our study is the first to identify the link between endothelial barrier function in the etiology of AAA. We observed that early and long‐lasting EC barrier dysfunction was involved in mouse AAA development and confirmed the involvement of EC barrier dysfunction in human aortic aneurysmal progression.

Ang II‐induced mouse aortas were used to scRNA‐seq, and we first confirmed that early and long‐lasting EC barrier dysfunction is involved in mouse abdominal aortic aneurysm. Human thoracic aortic dissection specimens and control samples were used to bulk mRNA‐seq that confirmed that endothelial barrier dysfunction is involved in aortic dissection progression, as documented by previous studies. These results showed that EC barrier dysfunction is not only a pathological change in the progression of thoracic aortic dissection but also is an early and long‐lasting pathological change involved in abdominal aortic aneurysm. However, the study has the limitation that human thoracic aortic specimens that we used may not be appropriate samples for the study because thoracic aortas and abdominal aortas may have different protein amounts and distributions due to variations in the structure, hemodynamic forces, extracellular matrix composition, smooth muscle cell phenotype, and pathological genetics.^[^ ^50^, ^51^ ^]^ Control specimens and aortic aneurysm from the abdominal aorta would be ideal.

In this study, we focused on ALDH2 as one upstream gene with single nucleotide polymorphisms (SNPs) in regulating endothelial barrier function. The Glu504Lys polymorphism of ALDH2 is seen in 8% of the world population and ≈30–50% of Asians. We previously showed that the pharmaceutical inhibitor Daidzin prevented vascular SMC phenotype switch and significantly reduced the incidence of aortic aneurysm and dissection.^[^ ^2^ ^]^ As the effect of Daidzin is not cell type‐specific, we combined data from ALDH2^−/−^ mice, ALDH2^−/−^ApoE^−/−^ mice, ALDH2^ECKO^PCSK9^OE^ mice and ALDH2^EC‐KD^ApoE^−/−^ mice to confirm the participation of ALDH2 in endothelial cells. Taking clues from the two studies, we conclude that ALDH2 has a dual impact in protecting two major cell types and highlight its vital role as a therapeutic target for AAA.

There are challenges to be overcome in the translational relevance of ALDH2: 1) ALDH2 has mainly protective roles in the heart and the cardiac vasculature, and we propose a tissue‐specific strategy of ALDH2 inhibition to improve risk management of cardiovascular disease.^[^ ^29^, ^52^, ^53^ ^]^ Patients with a high risk of developing AAA as opposed to ischemic heart disease (IHD) may likely benefit more from ALDH2 inhibition. On the other hand, ALDH2 inhibition may not be the best option for AAA patients with known IHD. 2) Since ALDH2 is a critical enzyme in alcohol oxidation and clearance of toxic aldehydes in cells, its deletion or knockdown, even if only in one specific cell type, might have unexpected deleterious consequences. Therefore, a compensation or co‐treatment strategy to maintain the detoxifying functions is needed when ALDH2 is knocked down for therapy of AAA.

The ELK3 gene encodes a member of the ETS‐domain transcription factor family and the ternary complex factor (TCF) subfamily that can regulate transcription when recruited by serum response factor to bind to serum response elements.^[^ ^39^ ^]^ Studies suggested that ELK3 is a strong transcriptional repressor due to the activity of two repressor domains, and it is able to be converted into a transcriptional activator by signal‐induced phosphorylation.^[^ ^54^, ^55^ ^]^ In this study, we examined the levels of phosphorylated ELK3 and total ELK3 and found that shALDH2 reversed Ang II‐induced down‐regulation of phosphorylated ELK3 and total ELK3 in HUVECs. These results confirmed that phosphorylated ELK3 functions as a transcriptional activator in HUVECs to upregulate expression of endothelial barrier markers. Moreover, the protective role of ELK3 in this study was consistent with previous publications, in which ELK3 was shown to upregulate VE‐cadherin expression in human pulmonary microvascular endothelial cells in endotoxin‐induced acute lung injury.^[^ ^56^ ^]^ However, the specific signals to phosphorylate ELK3 need to be further explored.

In addition, LIN28B was shown to upregulate downstream ELK3 expression. LIN28B is an RNA binding protein (RBP) and is associated with RNA splicing, localization, stability, degradation, and translation by binding to the mRNA or microRNA of target genes.^[^ ^42^, ^57^, ^58^ ^]^ Moreover, LIN28B is known to promote the development of neuroblastoma and colon cancer, among other neoplasias.^[^ ^59^, ^60^ ^]^ One possible therapeutic strategy is LIN28B inhibition, although there are no such approved drugs to date. However, it is possible that LIN28B inhibition may increase the risk of aortic aneurysm. Thereby, a routine CT screening should be considered by basic scientists and clinical practitioners. Most importantly, the newly developed magnetic resonance imaging (MRI) probes based on disturbed endothelial tight junction barrier function can predict the formation of thoracic aortic aneurysm and dissection. This non‐invasive imaging detection provides new options to identify AAA patients at early stages.^[^ ^23^ ^]^

We also intriguingly found that Daidzin treatment mitigated the interaction between ALDH2 and LIN28B. Daidzin binds in the hydrophobic pocket of ALDH2 without altering the protein structure.^[^ ^61^ ^]^ Furthermore, in silico structural analysis showed that the binding sites of this inhibitory compound at the substrate catalytic site were in proximity to LIN28B's binding site (both in its hydrophobic pocket), raising the possibility of a competitive binding effect between Daidzin and Lin28B. Nonetheless, the precise mechanisms are beyond the scope of this study and warrant further study.

In conclusion, findings from our study revealed a protective property of deficient ALDH2 against EC barrier dysfunction during early stages of AAA progression through a LIN28B‐ELK3‐mediated mechanism. Conversely, high ALDH2 levels in AAA tissues may accelerate the early progression of AAA. These findings favor a role for ALDH2 as a potential therapeutic target in the management of AAA.

## 4 Experimental Section

### Human Tissues Collection

This study was approved by the Medical Institutional Ethics Committee of Qilu Hospital, Shandong University, China. Written informed consent was provided by all participants or the organ donors’ legal representatives before enrollment.

Human thoracic aortic control samples were collected from six organ donors (male). Three organ donors’ samples were used to bulk mRNA‐seq and three samples were used for immunohistochemical validation. All donors were deceased due to acute trauma or cerebral hemorrhage. The range of donor ages was 37–75 years, with a median age of 51.0 years.

Human aortic dissection samples were obtained from six AAD patients undergoing open operative repair (three males and three females). These intimal tissues included four ascending aortas and two descending aortas and were used to bulk mRNA‐seq and for immunohistochemical validation. The aortas from controls and AAD patients were collected using the same technical conditions and were dissected carefully to reduce the possibility of cell damage as a consequence of the surgery. The range of AAD patients ages was 47–60 years, with a median age of 52.67 years. Information regarding these tissue samples is provided in Table S1, Supporting Information.

### Genetic Mouse Models

Three genetic mouse models were included in this study. The global ALDH2 knockout mice (ALDH2^−/−^) were constructed by University of Occupational and Environmental Health (Fukuoka, Japan) on a C57BL/6J (WT) background and backcrossed for at least ten generations with in‐house C57BL/6J mice to create a congenic strain.

ALDH2^−/‐^ mice were crossed with ApoE^−/−^ mice (from Beijing Viewsolid Biotech Animal Experimental Center) to generate ALDH2^−/−^ApoE^−/−^ mice. The ALDH2^flox^ mice containing floxP sites flanking exon 12 of ALDH2 and Tek‐creERT2 mice were generated on the C57BL/6J (WT) background at Beijing Viewsolid Biotech Animal Experimental Center. The ALDH2^ECKO^ mice were generated by crossbreeding ALDH2^flox^ mice with Tek‐creERT2 mice.

Sample size for each experiment is described in the figure legends section. All mouse manipulations were performed following the recommendations in the Guide for the Care and Use of Laboratory Animals published by the US National Institutes of Health (NIH publication no. 85–23 revised 1996) and approved by the Ethics Committee and the Scientific Investigation Board of Qilu Hospital, Shandong University, China.

### AAA Mouse Models

Because female mice were naturally resistant to Ang II treatment and present significantly lower AAA incidence, only male mice were included in the present study.

The PCSK9/Ang II‐induced murine AAA model was generated as previously described.^[^ ^33^ ^]^ In brief, 8–10 weeks male mice were administered via the lateral tail intravenous (IV) injections of 2 × 10^11^ genomic copies of adeno‐associated virus (AAV, serotype 8) carrying a gain‐of‐function mutation of the mouse Pcsk9 (AAV.Mpcsk9^D377Y^) and food was changed to a western diet (HCD, 17.3% protein, 21.2% fat, 48.5% carbohydrate, 0.2% cholesterol by mass, and 42% calories from fat; TD.88137, Envigo) immediately after injection. Two weeks after AAVs injection, Osmotic pumps (Durect, Alzet 2004) containing Angiotensin II (Ang II) (1000 ng kg^−1^ min^−1^, Sigma, 05‐23‐0101) were implanted and western diet was continued for 4 weeks to induce AAA formation.

The Ang II‐induced murine AAA model on ApoE^−/−^ background was generated using Osmotic pumps (Durect, Alzet 2004) containing either Ang II (1000 ng kg^−1^ min^−1^, Sigma, 05‐23‐0101) or saline for infusion for 8 weeks in ApoE^−/−^ male mice.

The endothelial injury model was established by Ang II (700 ng kg^−1^ min^−1^, Sigma, 05‐23‐0101) infusion for 2 weeks on C57BL/6J mice following the reported protocol.^[^ ^62^ ^]^

Euthanasia was performed by cervical dislocation when deep reflexes disappeared under 1.2–1.5 Vol.% isoflurane anesthesia after indicated times post minipump implantation. Abdominal aortas harvested were snap‐frozen in liquid nitrogen and then stored at −80 °C pending further processing.

### Statistical Analysis

Continuous data were expressed as means ± SEM, and categorical variables were expressed as number and percentage (%). Normality of data distribution was tested by using a Shapiro‐Wilks test, then the Brown–Forsythe test was performed to check for equal variances among normally distributed data. Comparisons between groups were performed by Student t‐test or one‐way ANOVA followed by Bonferroni post hoc test if the assumption of equal variances was met. Non‐parametric tests were used for data that were not normally distributed or the sample sizes were small. The Mann–Whitney test was applied for comparisons between two groups. The Kruskal–Wallis test with Dunn's multiple comparisons test were applied for comparisons among more than two groups. The detailed statistical analysis applied to each experiment is presented in the corresponding figure legends. Statistical significance was considered at *p* < 0.05. The “n” of experiments in the figure legends represents biological replicates. All data were analyzed using GraphPad Prism version 8.0.

Additional details are provided in Supporting Information.

## Conflict of Interest

The authors declare no conflict interest.

## Author Contributions

K.Y., S.C., and J.W. contributed equally to this work. K.Y., S.C., and J.W. acquired and analyzed the data and wrote the manuscript; T.X., H.Y., H.Y., J.G., J.Z., and Y.G. performed part of the experiments and acquired data; J.W. conducted the echocardiographic analysis; H.D., P.L., and Z.L. performed the bioinformatic analysis; X.Y., and C.Z., provided valuable suggestions about the experiment; C.P., J.P., and J.W. participated in result discussion. F.X. and Y.C. designed the study protocol and corrected the manuscript. All authors discussed the results and contributed to the final manuscript.

## Supporting information

Supporting Information

Supporting Information

Supporting Information

Supporting Information

Supporting Information

## Data Availability

The data that support the findings of this study are available from the corresponding author upon reasonable request.
