# Supplementary material for: Early Progression of Abdominal Aortic Aneurysm is Decelerated by Improved Endothelial Barrier Function via ALDH2‐LIN28B‐ELK3 Signaling
Source: Adv Sci (Weinh). 2023 Oct 11;10(32):2302231. doi: 10.1002/advs.202302231 (PMC10646281; doi:10.1002/advs.202302231)
Supplement: Supplementary file 1 — Supporting Information [file ADVS-10-2302231-s002.pdf]

ADVANCED SCIENCE
Open Access

Supporting Information

for *Adv. Sci.*, DOI 10.1002/advs.202302231

Early Progression of Abdominal Aortic Aneurysm is Decelerated by Improved Endothelial Barrier Function via ALDH2-LIN28B-ELK3 Signaling

Kehui Yang, Sumei Cui, Jingwen Wang, Tonghui Xu, Han Du, Hongwei Yue, Huaqing Ye, Jialin Guo, Jian Zhang, Pengpai Li, Yunyun Guo, Chang Pan, Jiaojiao Pang, Jiali Wang, Xiao Yu, Cheng Zhang, Zhiping Liu, Yuguo Chen and Feng Xu**

Supporting Information

Early Progression of Abdominal Aortic Aneurysm is Decelerated by Improved Endothelial Barrier Function via ALDH2-LIN28B-ELK3 Signaling

Kehui Yang[1, 2, 3†], Sumei Cui[1, 2, 3†], Jingwen Wang[1, 2, 3†], Tonghui Xu[1, 2, 3], Han Du[1, 2, 3], Hongwei Yue[1, 2, 3], Huaqing Ye[1, 2, 3], Jialin Guo[1, 2, 3], Jian Zhang[1, 2, 3], Pengpai Li[4], Yunyun Guo[1, 2, 3], Chang Pan[1, 2, 3], Jiaojiao Pang[1, 2, 3], Jiali Wang[1, 2, 3], Xiao Yu[5], Cheng Zhang[3, 6], Zhiping Liu[4], Yuguo Chen[1,2,3*], Feng Xu[1,2,3*]

[1] Department of Emergency Medicine, Chest Pain Center, Qilu Hospital of Shandong University; Jinan, Shandong 250012, China.

[2] Shandong Provincial Clinical Research Center for Emergency and Critical Care Medicine, Key Laboratory of Emergency and Critical Care Medicine of Shandong Province, Key Laboratory of Cardiopulmonary-Cerebral Resuscitation Research of Shandong Province, Shandong Provincial Engineering Laboratory for Emergency and Critical Care Medicine, Qilu Hospital of Shandong University; Jinan, Shandong 250012, China.

[3] The Key Laboratory of Cardiovascular Remodeling and Function Research, Chinese Ministry of Education, Chinese Ministry of Health and Chinese Academy of Medical Sciences; The State and Shandong Province Joint Key Laboratory of Translational Cardiovascular Medicine; Qilu Hospital of Shandong University; Jinan, Shandong 250012, China.

[4] Department of Biomedical Engineering, School of Control Science and Engineering, Shandong University; Jinan, Shandong 250012, China.

[5] Key Laboratory Experimental Teratology of the Ministry of Education and Department of Physiology and Pathophysiology, School of Basic Medical Sciences, Shandong University; Jinan, Shandong 250012, China.

[6] Department of Cardiology, Qilu Hospital of Shandong University; Jinan, Shandong 250012, China.

[†]These authors contributed equally to this work.

*Corresponding author. Email: Feng Xu, xufengsdu@126.com, xufengsdu@email.sdu.edu.cn; Yuguo Chen, chen919085@sdu.edu.cn.

This file includes:
Supplementary Materials and Methods
Supplementary Figure 1-14 and Figure legends
Supplementary table 1-3

Materials and Methods

Bulk mRNA-seq of human samples

Total RNA was extracted with Trizol and assessed with the kaiaoK5500®Spectrophotometer (Kaiao, Beijing, China) and the RNA Nano 6000 Assay Kit of the Bioanalyzer 2100 system (Agilent Technologies, CA, USA). Total RNA samples that met the following requirements were used in subsequent experiments: RNA integrity number (RIN) > 7.0 and a 28S:18S ratio > 1.8. The sequencing quality was assessed with FastQC (Version 0.11.5) and then low-quality data were filtered using NGSQC (v0.4), a quality value of over 50% bases of the read was less than 5, and trimmed the adapter sequence, and removed the reads containing poly-N using in-house Perl scripts. Sequence libraries were generated and sequenced by CapitalBio Technology (Beijing, China). RNA from 6 human AAD intimal tissues and 3 control intimal sample was extracted for RNA sequence study. The differential genes list is available in Excel S1, Supporting Information.

Echocardiography

After Ang II infusion, the maximal diameter of the abdominal aorta was measured with a high-resolution ultrasound imaging system (VisualSonics, Vevo3100) in a double-blind way. Aneurysm incidence was quantified based on a definition of an external width of the suprarenal aorta that was increased by 50% or greater compared with aortas from saline-infused mice. The ruptured aorta was excluded from the analysis of aortic diameter but included in the analysis of aneurysm incidence. Mice were anesthetized with 1.2-1.5 Vol % isoflurane.

Single-cell RNA sequencing

Regular digestion of mouse aorta samples. The methods of the aortic aneurysm mouse model are as described before. Aortas were harvested at day 0 without Ang II exposure, as well as 3 and 28 days after Ang II exposure. Preparation of the single-cell suspension of the aortic cells was performed following a previously described enzymatic digestion protocol[1]. Briefly, the isolated whole aorta pooled from five mice in each group were cut and digested with an enzyme solution (450 U/mL collagenase type I, 125 U/mL collagenase type XI, 60 U/mL hyaluronidase type I-s, and 60 U/mL DNase I) for 1.5 hours at 37°C. The cell suspension was strained

through a 30 μm filter and washed twice with PBS. The cells were resuspended in 0.4% BSA-PBS and the cell viability was greater than 80%, as confirmed by trypan blue staining. The resuspended cells were subjected to scRNA-seq.

Cell filtering. Dimensional reduction, clustering, and analysis of single-cell RNA sequencing data were performed using the R package Seurat. Cells were filtered out if more than 4,000 genes or less than 200 genes were detected, the percentage of mitochondrial gene counts were higher than 25% and hemoglobin genes counts were higher than 10%.

Single-cell clustering. Clustering was conducted using Seurat (v.4.1.1). To avoid batch effects among samples and experiments, we used Harmony to integrate the samples. We used the FindClusters function of Seurat on the basis of the shared nearest neighbor (SNN-cliq) clustering method with parameters (k = 20, dimuse = 1:20, res = 0.1). The Seurat functions DotPlot were used to visualize the gene expression with dot plot.

Definition of marker genes of cell groups. Marker genes for each cluster were determined using the Wilcoxon rank-sum test via the FindAllMarkers function in Seurat. The FindAllMarkers function was used with the parameters min.pct=0.25, logfc.threshold=0.25, only.positive=TRUE and return.thresh=0.01. Based on the known marker genes, cell types were assigned to each cluster, including smooth muscle cells, fibroblasts, macrophages, ECs, T cells, B cells, granulocyte cells, proliferating cells and adipocytes.

Endothelial cluster gene set signatures. The FindAllMarkers Seurat function was utilized to find positive markers of EC population using the Wilcoxon rank-sum test. Pathway enrichment analysis was performed using enrichKEGG function to identify gene sets in clusterProfiler (V4.4.4). The differential genes list versus between day 28 and day 0 is available in Excel S2, Supporting Information. The differential genes list versus between day 3 and day 0 is available in Excel S3, Supporting Information.

Cell cultures

Different cell lines were chosen according to different purposes.

Primary Human Aortic Endothelial Cells (HAECs) and Primary Human Umbilical Vein Endothelial Cells (HUVECs) were purchased from ScienCell Research Laboratories, which were cultured in Endothelial Cell Medium (ScienCell, 1001). The HUVEC cell line were purchased from Pricella and maintained with RPMI 1640 medium (Sigma, 8758). Primary HAECs, primary HUVECs and the HUVEC cell line were used to determine endothelial barrier function.

HEK293T cells were purchased from the American Type Culture Collection (Manassas, VA, USA) and were cultured in high-glucose DMEM supplemented with 10% FBS, 100 units/mL penicillin and 100 μg/mL streptomycin. HEK293T cells were used in cell biology research in this research, because of their reliable growth and propensity for transfection.

siRNA-mediated gene knockdown

ELK3-siRNA, FOS-siRNA, TEAD1-siRNA, LIN28B-siRNA, and scramble-siRNA were purchased from GenePharma (Shanghai, China). Lipofectamine RNAiMAX reagents (Thermo Fisher Scientific,13778150) were used for transient transfection of siRNA duplexes into HUVECs cell line. Sequences of siRNAs are available in Table S2, Supporting Information. After 24 hours of transfection, cells were ready for further experiments.

Plasmid construction and transfection

The human ALDH2, ELK3 and LIN28B cDNA was cloned into pcDNA3.1 by Biosune biotechnology. Desired DNA fragments of the human JAM-A、claudin5、VE-cadherin and ELK3 promoter were PCR-amplified and cloned into the pGL3-Basic luciferase reporter vector (Promega, E1751). All constructs were confirmed by DNA sequencing. Lipofectamine 2000 reagents (Thermo Fisher Scientific, 11668030) were used for transfection of plasmids into HUVEC cell line or HEK293T.

Construction of adeno-associated virus

AAV serotype 1 (AAV1) vector serotypes (with an ICAM2 promoter) is the preferred viral vector to target aortic endothelial cell after injection. AAV1 shRNA ALDH2 sequence was cloned into the pAV-ICAM2-GFP-mir30-shRNA to get ALDH2-RNAi plasmid[2]. AAV1 shRNA ELK3 sequence was cloned into the pAV-ICAM2-GFP-mir30-shRNA to get an ELK3-RNAi plasmid. Sequences of shRNA are available in Table S2, Supporting Information. AAV1 ELK3 sequence was cloned into the pAV-ICAM2-P2A-GFP to get an ELK3 overexpression plasmid. Specific sequences and nonspecific controls were separately constructed in one vector, and AAV1 harboring these sequences were generated by Vigene Biosciences (Jinan, China). Mice were injected with one of the above viruses (5×10^{11} pfu/kg) for 4 weeks before they were randomly grouped and infused with Ang II.

Real-time quantitative PCR (RT-qPCR)

Tissues or cells were mechanically homogenized and total RNA was extracted using Trizol Reagent (Sigma, T9424). Reverse transcription into cDNA was performed using the Reverse Transcription System kit (Vazyme, R223-01). The synthesized cDNA was amplified with a standard quantitative polymerase chain reaction (qPCR) protocol including the use of ChamQ Universal SYBR qPCR Master Mix (Vazyme, Q711-02). Amplification products were normalized against GAPDH mRNA, which was amplified in the same reaction as an internal control. Data were analyzed according to the $2^{-\Delta\Delta Ct}$ method. The fold change in mRNA expression was quantified relative to control. RT-qPCR primers are listed in Table S3, Supporting Information.

Western blot

Tissue or cell lysates with the same protein content (determined by the BCA method; Boster, AR0146) were prepared. Proteins were separated by 10% SDS-PAGE or 12% SDS-PAGE and transferred to polyvinylidene difluoride (PVDF) membranes. After being blocked for 1.5 hours in 5% non-fat milk, the bands were incubated overnight at 4°C with primary antibodies. After washing, secondary antibodies were incubated for

1.5 hours. The bands were scanned and detected by a chemiluminescence instrument (General Electric Company, AI600RGB). The relative intensity of immunoreactive bands was assessed by Image J software. The results were normalized to GAPDH levels and expressed as % of control. All experiments were repeated at least thrice. The following primary antibodies were used: anti-VE-cadherin (Abcam, ab33168), anti-ALDH2 (Abcam, ab133306), anti-JAM-A (Santa Cruz Biotechnology, sc-53623), anti-claudin-5 (Santa Cruz Biotechnology, sc-374221 and Abcam, ab131259), anti-vinculin (Santa Cruz Biotechnology, sc-73614) and anti-α-catenin (Santa Cruz Biotechnology, sc-9988), anti-β-catenin (Santa Cruz Biotechnology, sc7963), anti-p-β-catenin (Santa Cruz Biotechnology, sc57535), anti-γ-catenin (Santa Cruz Biotechnology, sc-514115)， anti-p120-catenin (Santa Cruz Biotechnology, sc-23873), anti-paxillin (Santa Cruz Biotechnology, sc-365379), anti-p-paxillin (Santa Cruz Biotechnology, sc-365020), anti-ELK3(Santa Cruz Biotechnology, sc-134401), anti-phospho-ELK3 (Ser357) (Thermo Fisher Scientific, PA5-40287), anti-LIN28B (Abcam, ab19188) and anti-GAPDH (Abcam, ab181602).

Immunoprecipitation (IP) and Co-immunoprecipitation (Co-IP) assays

For IP in HUVEC cell line, cells were washed thrice and scraped into ice-cold PBS. The pellets were re-suspended with lysis buffer. The protein supernatant fluid was respectively incubated with 2 μg of antibody against LIN28B (Abcam, ab19188) and rabbit IgG separately at 4 °C overnight incubation and rotation. The immune complexes were incubated with 40 μL of protein A/G sepharose beads for 3 hours rotation and washed with the lysis buffer for 3 times. 20 μL of 4×SDS loading buffer was added into each sample, which was then subjected to western blot analysis. High-resolution mass spectrometry-based proteomics was performed to determine proteins interact with LIN28B. One hundred and seventy-eight proteins were identified in the protein mixture enriched by LIN28B antibody and KEGG enrichment analysis was generated by Metascape (https://metascape.org). Annotation and enrichment results are listed in Excel S4, Supporting Information.

For Co-IP, cells were lysed with IP lysis buffer. The primary antibody was coupled with protein samples and incubated at 4°C overnight. Add Protein A/G beads to each sample and incubate the lysate beads mixture at 4°C under rotary agitation for 3 hours. After IP, the samples were washed with IP lysis buffer thrice. Proteins were eluted with 1x SDS sample buffer.

ChIP-X enrichment analysis

The ChIP-X Enrichment Analysis 3 (ChEA3) tool (https://amp.pharm.mssm.edu/ChEA3) was used to predict transcription factors (TFs) as potential EC barrier regulators. Firstly, submit a query with EC barrier regulator genes in the query text box (CDH5, CLDN5, VCL, F11R, CTNND1, PXN, CTNNA1, CTNNB1, JUP, CDH1, CDH5, PTK2, SRC, RAC1, NOS3, TJP1, PTPRB, ESAM, PECAM1, VCAM1, JAM2, JAM3) and the MeanRank table is shown in the ChEA3 benchmark. Secondly, according to TF ranking and results of the table, we chose the top 25 TFs whose putative transcriptional targets are most closely similar to the query set. Thirdly, these 25 TFs were experimentally verified in aortic tissue and HUVEC cell line.

Chromatin immunoprecipitation (ChIP) assay

HUVEC cell line transfected with vector or ELK3 plasmid followed by serum starvation for 24 hours, were first incubated with 1% formaldehyde for 15 minutes to cross-link DNA protein complexes. Cells were then rinsed, harvested and lysed. Cell lysates were sonicated to produce genomic DNA fragments 300-1000 bp in length. The lysates were pre-cleaned with a mixture of salmon sperm DNA-protein A/G, followed by immunoprecipitation with an anti-ELK3 (Novus Biologicals, NBP1-83960) or normal IgG (Novus Biologicals, NBP2-24891) antibody overnight at 4°C. Then, single-strand salmon sperm DNA saturated with protein A/G plus-agarose beads (Santa Cruz Biotechnology, sc-2003) was added and incubated for 1 hours. The immunocomplex beads were then washed sequentially with wash buffer and TE buffer and eluted with elution buffer (100 mM $NaHCO_3$, 1% SDS). The eluted immunocomplexes were incubated with proteinase K

buffer at 65°C to release the cross-linked DNA, which was further purified by phenol/chloroform/isoamyl alcohol and amplified by PCR to measure the ELK3-bound sequences.

RNA immunoprecipitation (RIP) assay

The RNA isolated from HUVEC cell line was incubated in immunoprecipitation buffer (150 nmol/L NaCl, 0.1% NP-40, 10 mmol/L Tris-HCl, pH 7.4) containing anti-LIN28B antibody (Abcam, ab191881) and IgG (Cell Signaling Technology, 2729) at 4 °C for 12 hours. Then, magnetic protein A/G beads was added and continuously incubated at 4 °C for 2 hours. After being centrifuged five times and washed with IP buffer, the LIN28B-bound RNA was enriched and subjected to RT-qPCR analysis.

Dual-luciferase reporter assay

The plasmids pcDNA3.1-ELK3, pcDNA3.1-ALDH2, pGL3-JAM-A-luc, pGL3-claudin-5-luc and pGL3-VE-cadherin-luc were purchased from GenePharma. HUVEC cell line were transiently co-transfected with luciferase reporter and Renilla plasmid by using Lipofectamine 2000 reagent according to the manufacturer's instructions. After 48 hours of transfection, cells were harvested for measurement of luciferase activity by using a luciferase assay system (Promega, E1910).

Measurement of mRNA stability

To measure the stability of ELK3 mRNA, HUVEC cell line were pretreated with actinomycin D (5 μg/mL, MedChemExpress, HY-17559) for 30 minutes. Total RNA of cells was then collected at 0, 4, 8, 12, and 24 hours after actinomycin D treatment, followed by RT-qPCR analysis using primers for the ELK3 coding sequence. The primers for RT-qPCR are listed in Table S3, Supporting Information.

***In vivo* Miles permeability assay**

On day 7, 14 or 28 post-AAA surgery, mice were injected with 100 μl of 1% Evans blue solution in 1× PBS through tail vein injection. One hour later, mice were perfused with saline in the left ventricle to remove intravascular Evans blue dye, and the heart and aorta tissues were excised. Then the surrounding adipose

tissues were removed, and images of the whole aorta were captured using a digital camera. The aortic cross-sections were mounted by Prolong Gold Antifade Reagent with DAPI and used for imaging. Due to the autofluorescence characteristic of Evans blue (excitation at 620 nm, emission at 680 nm), the amount of Evans blue that permeated into the aortic wall was visualized (red) using the fluorescent microscope as mentioned above.

Immunofluorescence and confocal microscopy

After being washed with PBS thrice, the cells coverslip or tissue section were fixed with 4% paraformaldehyde in PBS for 15 minutes at room temperature. Aspirate fixative, rinse three times in PBS for 5 minutes each. Permeabilize samples with 0.1% Triton X-100 in PBS for 10 minutes. Then the cells coverslip or tissue section were blocked with 5% bovine serum albumin for 30 minutes and then incubated with the following primary antibodies at 4°C overnight: anti-VE-cadherin (Abcam, ab33168), anti-p120-catenin (Santa Cruz Biotechnology, sc-23873) and anti-claudin-5 (Santa Cruz Biotechnology, sc-374221). Subsequently, the cells coverslip or tissue section were incubated with the appropriate secondary antibodies at room temperature for one hour. Finally, the cells coverslip or tissue section were mounted with DAPI. Images of cells were taken under the confocal laser scanning fluorescence microscopy (LSM710; Carl Zeiss AG, Jena, Germany).

Histological analysis

The entire aorta was fixed with 10% formalin for 24 hours at room temperature before being embedded in paraffin for sectioning. Tissues were sectioned at 5 μm and underwent H&E staining, Elastic tissue fibers-Verhoeff's Van Gieson (EVG) staining, Masson staining or immunohistochemical staining. After heat-mediated antigen retrieval, paraffin sections were blocked with 5% BSA and subjected to immunohistochemical staining. The sections were incubated overnight at 4°C with primary antibodies against ALDH2 (Abcam, ab133306) and ELK3 (Santa Cruz Biotechnology, sc-134401). After several washes with PBS, the sections were incubated with biotin-labeled goat anti-rabbit and goat anti-mouse secondary

antibodies at room temperature for 1 hours. Labeling was visualized with a 0.05% diaminobenzidine solution, and images of cells were taken under the confocal laser scanning fluorescence microscopy (Carl Zeiss AG, LSM710).

References

[1] A. S. Kalluri, S. K. Vellarikkal, E. R. Edelman, L. Nguyen, A. Subramanian, P. T. Ellinor, A. Regev, S. Kathiresan, R. M. Gupta, *Circulation* **2019**, 140, 147.
[2] B. Velasco, J. R. Ramírez, M. Relloso, C. Li, S. Kumar, J. P. Lopez-Bote, F. Pérez-Barriocanal, J. M. López-Novoa, P. J. Cowan, A. J. d'Apice, C. Bernabéu, *Gene Ther* **2001**, 8, 897.

Supplementary Figure legends

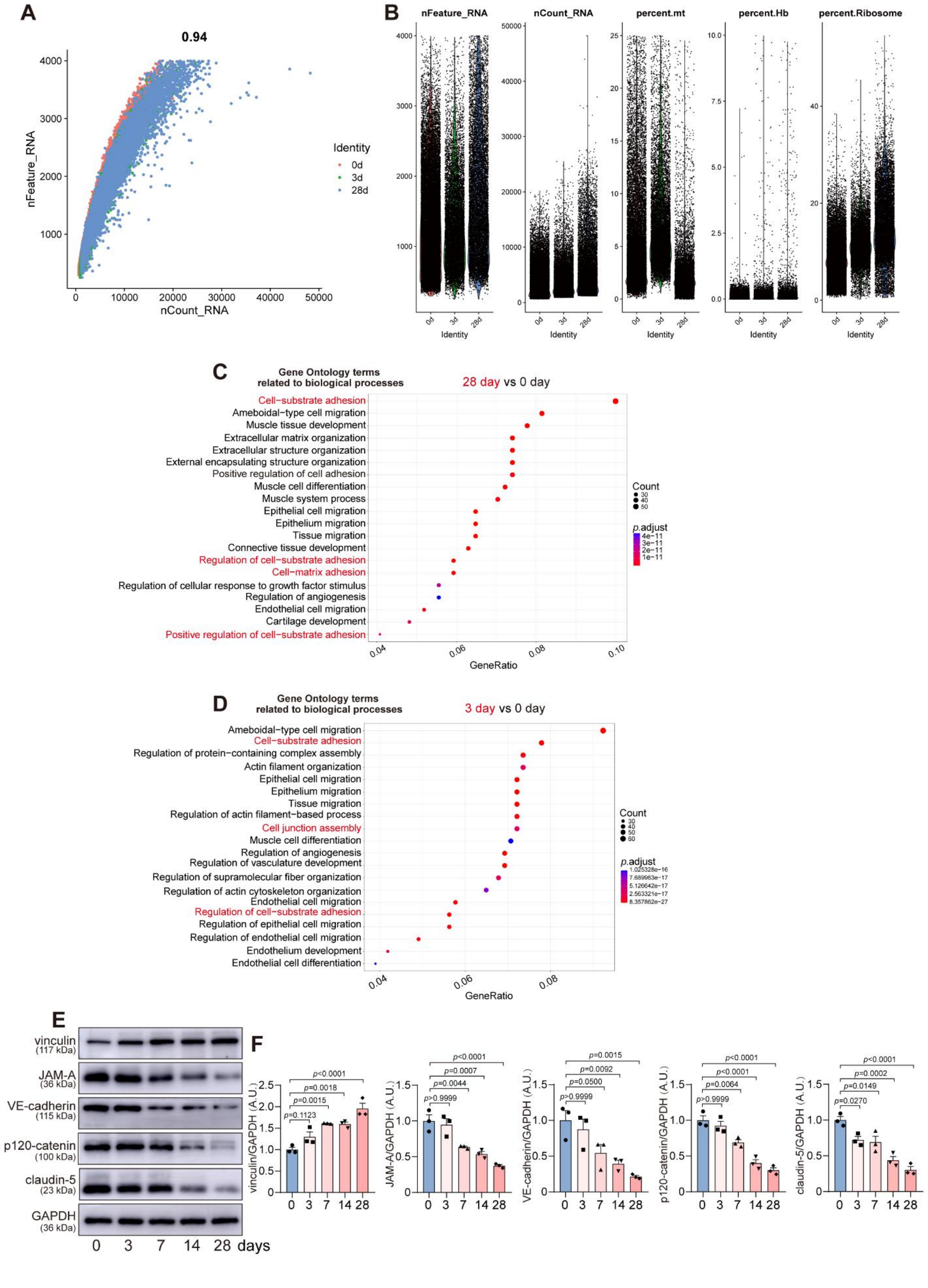
A
0.94
nFeature_RNA
nCount_RNA
Identity
0d
3d
28d
B
nFeature_RNA
nCount_RNA
percent.mt
percent.Hb
percent.Ribosome
Identity
C
Gene Ontology terms related to biological processes
28 day vs 0 day
Cell−substrate adhesion
Ameboidal−type cell migration
Muscle tissue development
Extracellular matrix organization
Extracellular structure organization
External encapsulating structure organization
Positive regulation of cell adhesion
Muscle cell differentiation
Muscle system process
Epithelial cell migration
Epithelium migration
Tissue migration
Connective tissue development
Regulation of cell−substrate adhesion
Cell−matrix adhesion
Regulation of cellular response to growth factor stimulus
Regulation of angiogenesis
Endothelial cell migration
Cartilage development
Positive regulation of cell−substrate adhesion
GeneRatio
Count
p.adjust
D
Gene Ontology terms related to biological processes
3 day vs 0 day
Ameboidal−type cell migration
Cell−substrate adhesion
Regulation of protein−containing complex assembly
Actin filament organization
Epithelial cell migration
Epithelium migration
Tissue migration
Regulation of actin filament−based process
Cell junction assembly
Muscle cell differentiation
Regulation of angiogenesis
Regulation of vasculature development
Regulation of supramolecular fiber organization
Regulation of actin cytoskeleton organization
Endothelial cell migration
Regulation of cell−substrate adhesion
Regulation of epithelial cell migration
Regulation of endothelial cell migration
Endothelium development
Endothelial cell differentiation
GeneRatio
Count
p.adjust
E
vinculin (117 kDa)
JAM-A (36 kDa)
VE-cadherin (115 kDa)
p120-catenin (100 kDa)
claudin-5 (23 kDa)
GAPDH (36 kDa)
0 3 7 14 28 days
F
vinculin/GAPDH (A.U.)
p=0.1123
p=0.0015
p=0.0018
p<0.0001
JAM-A/GAPDH (A.U.)
p>0.9999
p=0.0044
p=0.0007
p<0.0001
VE-cadherin/GAPDH (A.U.)
p>0.9999
p=0.0500
p=0.0092
p=0.0015
p120-catenin/GAPDH (A.U.)
p>0.9999
p=0.0064
p<0.0001
p<0.0001
claudin-5/GAPDH (A.U.)
p=0.0270
p=0.0149
p=0.0002
p<0.0001

Figure S1: Quality Control (QC) plots. (A) The number above the figure is the Pearson correlation coefficient between the number of UMI and genes in three samples. The closer the correlation coefficient was to 1, the stronger the correlation was. (B) The distribution of the number of genes, number of counts, percentage of mitochondrial fraction, percentage of hemoglobin genes fraction and percentage of ribosome fraction in three samples were showed by violin plots. Cells were filtered out if more than 4,000 genes, less than 200 genes were detected, the percentage of mitochondrial gene counts higher than 25% and hemoglobin genes counts higher than 10%. (C) A list of top 20 downregulated pathways from analysis of gene ontology terms related to biological processes in endothelial cells on day 28 versus day 0. (D) A list of top 20 downregulated pathways from analysis of gene ontology terms related to biological processes in endothelial cells on day 3 versus day 0. (E-F) Representative immunoblots and quantification for vinculin, JAM-A, VE-cadherin, p120-catenin and claudin-5 in aortas from ApoE$^{-/-}$ mice at serial time points of Ang II infusion (n=3 per group). Data are presented as mean ± SEM. One-way ANOVA followed by Bonferroni post hoc test was applied in (F). A.U. indicates arbitrary unit.

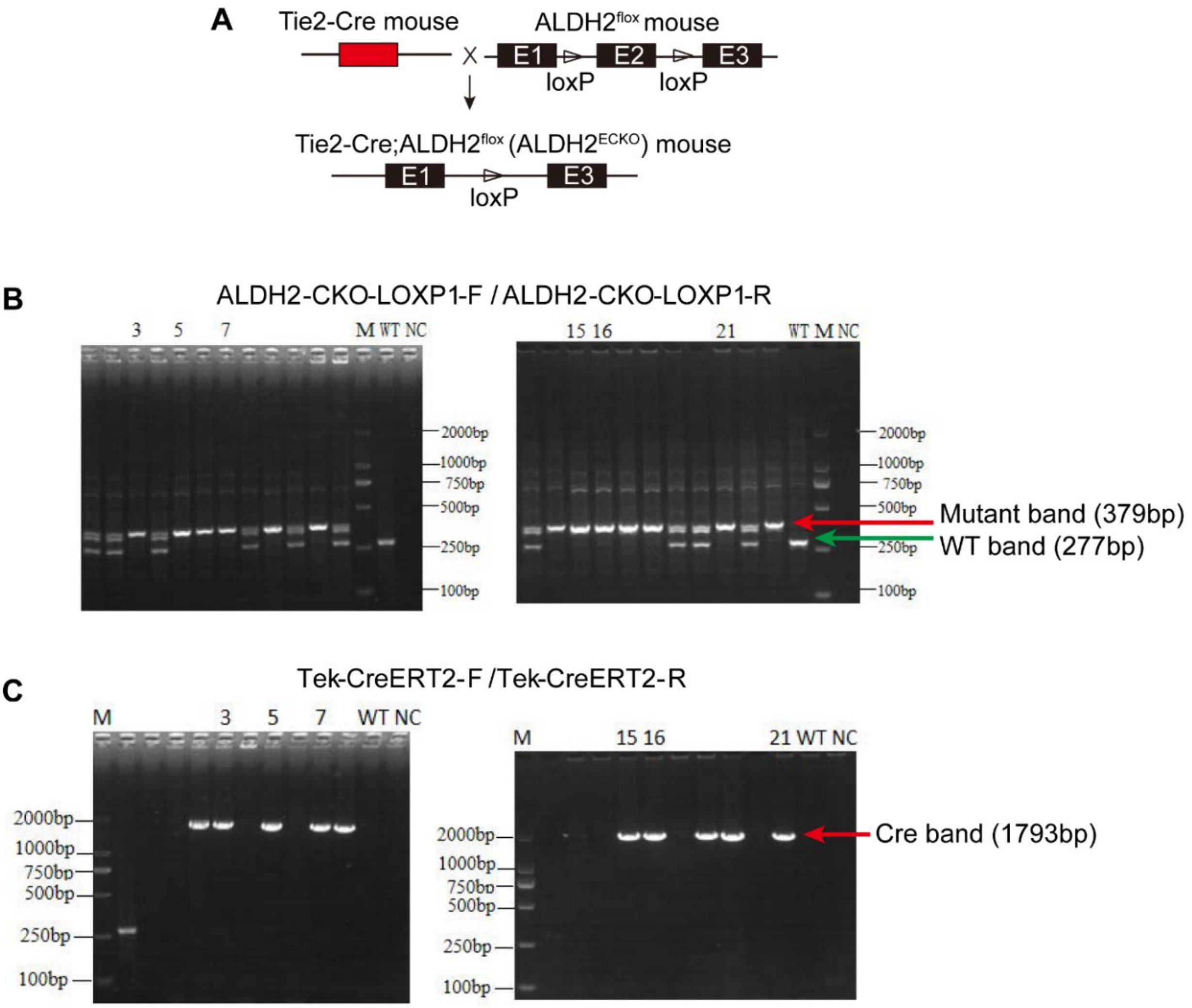

Figure S2: Construction of ALDH2^{ECKO} mouse and genotype identification. (A) Overview of EC-specific ALDH2 knockout (ALDH2^{ECKO}) mouse construction. (B) Mouse genotype identification. PCR analysis of ALDH2^{flox} mice. The red arrow refers to loxp (Mutant) band. The green arrow refers to wild type (WT) band. (C) Mouse genotype identification. PCR analysis of Tek-CreERT2 mice. The red arrow refers to Cre band.

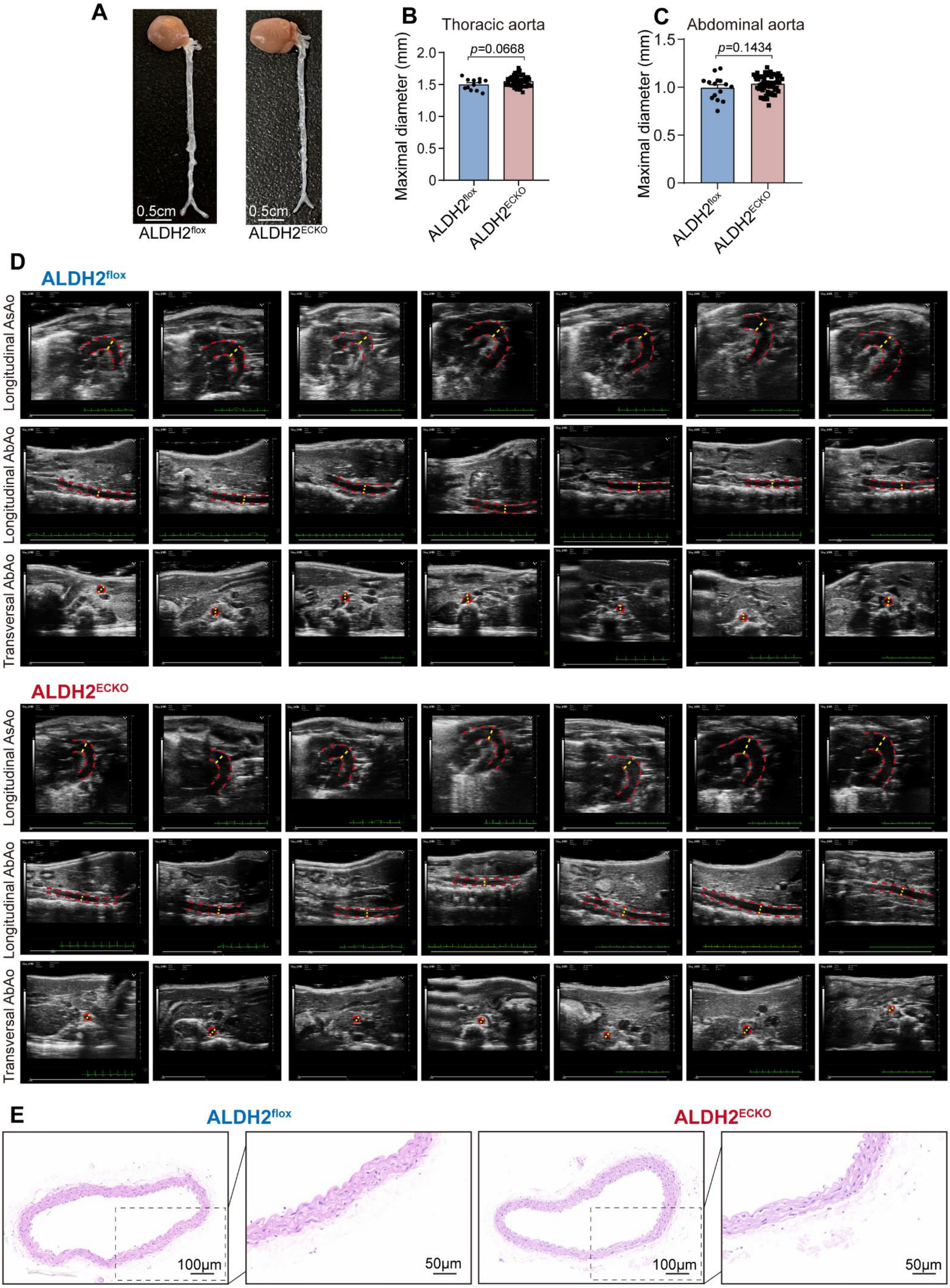
A
0.5cm
0.5cm
ALDH2flox
ALDH2ECKO
B
Thoracic aorta
p=0.0668
Maximal diameter (mm)
2.0
1.5
1.0
0.5
0
ALDH2flox
ALDH2ECKO
C
Abdominal aorta
p=0.1434
Maximal diameter (mm)
1.5
1.0
0.5
0
ALDH2flox
ALDH2ECKO
D
ALDH2flox
Longitudinal AsAo
Longitudinal AbAo
Transversal AbAo
ALDH2ECKO
Longitudinal AsAo
Longitudinal AbAo
Transversal AbAo
E
ALDH2flox
ALDH2ECKO
100μm
50μm
100μm
50μm

Figure S3: Vascular baseline characteristics for ALDH2^{flox} and ALDH2^{ECKO} mice. (A) Representative macroscopic images between ALDH2^{flox} and ALDH2^{ECKO} mice. Scale bar, 0.5cm. (B) Maximal thoracic aortic diameter between ALDH2^{flox} and ALDH2^{ECKO} mice. ALDH2^{flox}, n=12; ALDH2^{ECKO}, n=59. (C) Maximal abdominal aortic diameter between ALDH2^{flox} and ALDH2^{ECKO} mice. ALDH2^{flox}, n=15; ALDH2^{ECKO}, n=59. (D) Representative ascending aortic (AsAo) and abdominal aorta (AbAo) ultrasound images in ALDH2^{flox} and ALDH2^{ECKO} mice. (E) Representative H&E staining images of the abdominal aorta from ALDH2^{flox} and ALDH2^{ECKO} mice. Low-magnification images in E show the entire vascular wall at the site of analysis. Scale bar of low-magnification images in E, 100μm. Scale bar of high-magnification images in E, 50μm. Data are presented as mean ± SEM. Unpaired two-tailed Student's t tests were used in (B), (C).

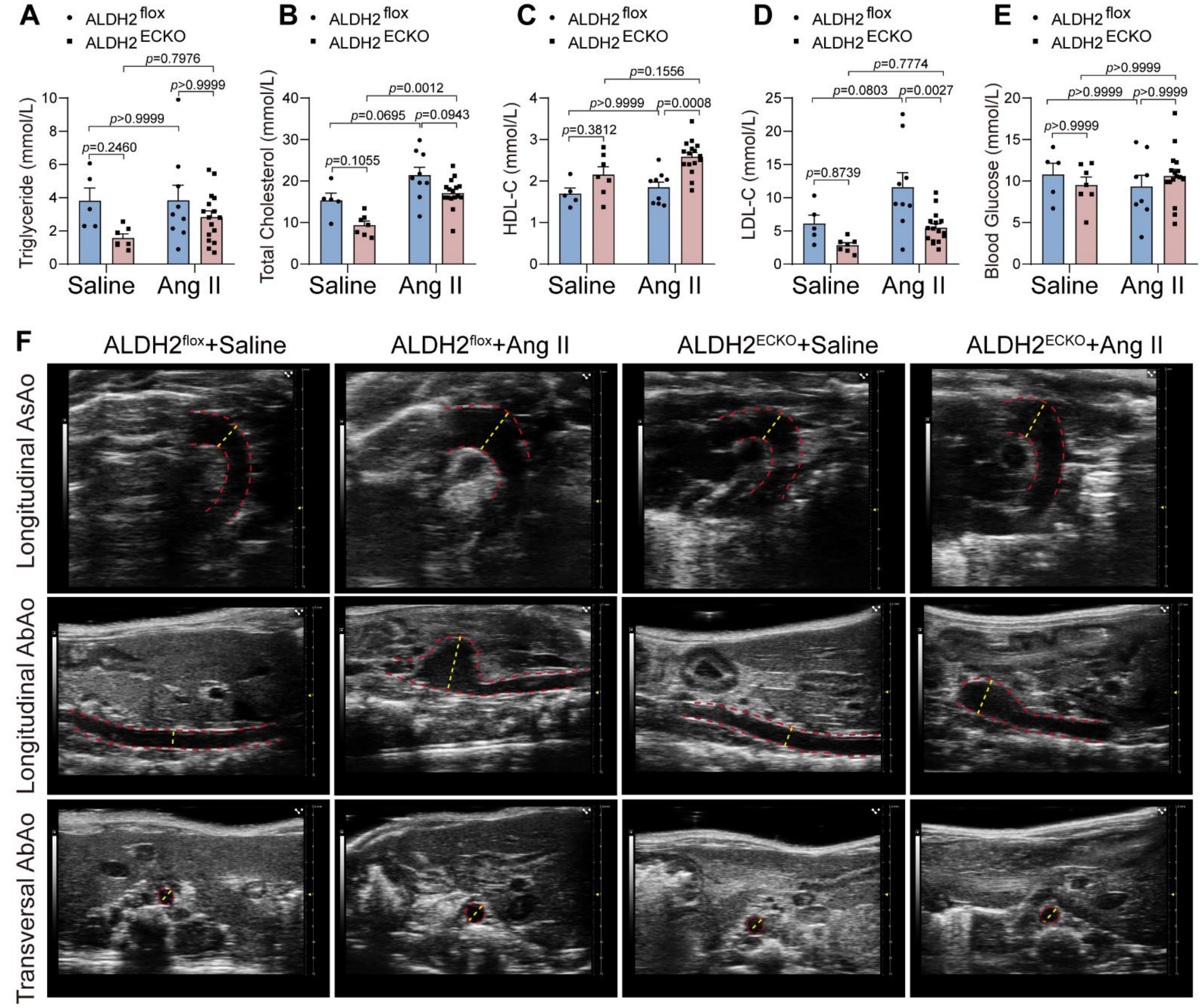

Figure S4: (A-E) Effect of ECs-specific ALDH2 knockout on Ang II-induced changes in plasma lipid profiles. (A) Triglyceride levels, (B) Total cholesterol levels, (C) High-density lipoprotein cholesterol (HDL-C) levels, (D) Low-density lipoprotein cholesterol (LDL-C) levels, and (E) Blood glucose levels. A through E,

ALDH2^{flox} +Saline, n=5; ALDH2^{ECKO}+Saline, n=7; ALDH2^{flox} + Ang II, n=9; and ALDH2^{ECKO}+Ang II, n=15. (F) Representative ascending aortic (AsAo) and abdominal aorta (AbAo) ultrasound images in 4 animal groups at 4 weeks following Ang II infusion. Data are presented as mean ± SEM. Two-way ANOVA followed by Bonferroni post hoc analyses were applied in (A-E).

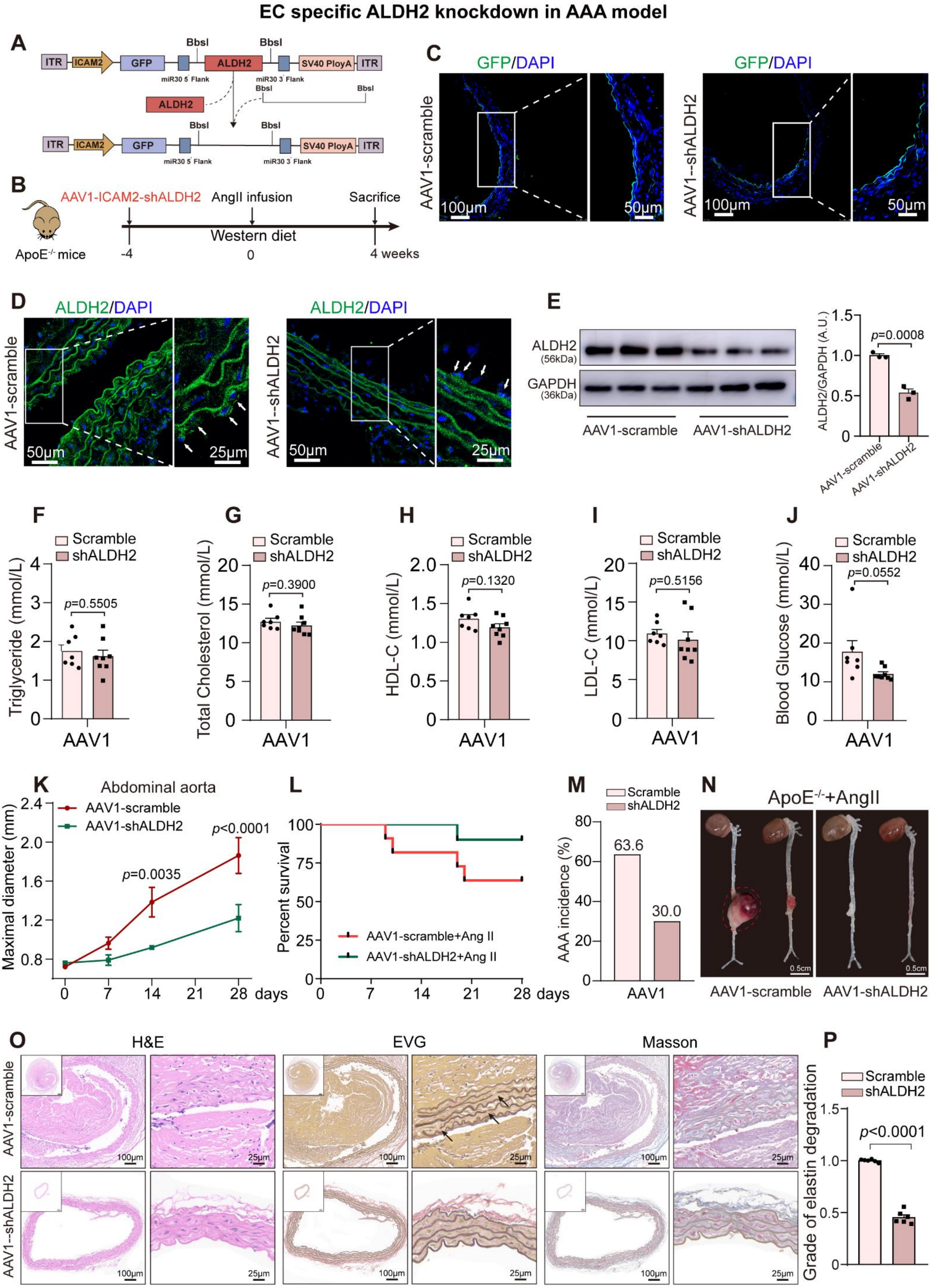

EC specific ALDH2 knockdown in AAA model
A
ITR
ICAM2
GFP
BbsI
ALDH2
SV40 PloyA
miR30 5' Flank
miR30 3' Flank
B
AAV1-ICAM2-shALDH2
AngII infusion
Sacrifice
Western diet
ApoE-/- mice
-4
0
4 weeks
C
GFP/DAPI
AAV1-scramble
AAV1--shALDH2
100μm
50μm
D
ALDH2/DAPI
25μm
E
ALDH2 (56kDa)
GAPDH (36kDa)
AAV1-scramble
AAV1-shALDH2
ALDH2/GAPDH (A.U.)
p=0.0008
F
Scramble
shALDH2
Triglyceride (mmol/L)
p=0.5505
AAV1
G
Total Cholesterol (mmol/L)
p=0.3900
H
HDL-C (mmol/L)
p=0.1320
I
LDL-C (mmol/L)
p=0.5156
J
Blood Glucose (mmol/L)
p=0.0552
K
Abdominal aorta
AAV1-scramble
AAV1-shALDH2
Maximal diameter (mm)
p=0.0035
p<0.0001
days
L
Percent survival
AAV1-scramble+Ang II
AAV1-shALDH2+Ang II
M
AAA incidence (%)
63.6
30.0
N
ApoE-/-+AngII
0.5cm
O
H&E
EVG
Masson
P
Grade of elastin degradation
p<0.0001

Figure S5: Knockdown of ALDH2 (aldehyde dehydrogenase 2) in aortic endothelial cells suppresses aortic dilation at an early stage and ultimately prevents the formation of AAA. (A) Adeno-associated-virus (AAV1 shRNA ALDH2 sequence was cloned into the pAV-ICAM2-GFP-mir30-shRNA to get AAV1-shALDH2 plasmids. (B) Schematic protocol: AAV1-shALDH2 or AAV1-scramble was injected prior to Ang II or saline infusion in mice for 4 weeks. (C) Viral GFP fluorescence in the aortic intima following tail vein injection of ICAM2 promoter-driven AAV1 carrying ALDH2-shRNA or scramble-shRNA. Scale bar, 100μm and 50μm. (D) Representative images of immunofluorescence staining of ALDH2 in the aortic intima following tail vein injection of ICAM2 promoter-driven AAV1 carrying ALDH2-shRNA or scramble-shRNA. Scale bar, 50μm and 25μm. (E) ALDH2 expression in intima by western blot following tail vein injection of ICAM2 promoter-driven AAV1 carrying ALDH2-shRNA or scramble-shRNA. (F-J) Effect of shALDH2 on Ang II-induced changes in plasma lipid profiles. (F) Triglyceride levels, (G) Total cholesterol levels, (H) High-density lipoprotein cholesterol (HDL-C) levels, (I) Low-density lipoprotein cholesterol (LDL-C) levels, and (J) Blood glucose levels. F through J, AAV1-scramble, n=7; AAV1-shALDH2, n=8. (K) Maximal aortic diameter between AAV1-scramble group and AAV1-shALDH2 group. Day 0: AAV1-scramble, n=15; AAV1-shALDH2, n=15. Day 7: AAV1-scramble, n=10; AAV1-shALDH2, n=10. Day 14: AAV1-scramble, n=7; AAV1-shALDH2, n=7. Day 28: AAV1-scramble, n=7; AAV1-shALDH2, n=7. (L) Survival curves in indicated groups. AAV1-scramble, n=11; AAV1-shALDH2, n=10. (M) AAA incidence between AAV1-scramble group and AAV1-shALDH2 group. AAV1-scramble, n=11; AAV1-shALDH2, n=10. (N) Representative macroscopic images between AAV1-scramble group and AAV1-shALDH2 group. Scale bar, 0.5cm. (O) Representative H&E, EVG staining and Masson staining of the mouse abdominal aorta between AAV1-scramble group and AAV1-shALDH2 group at 4 weeks following Ang II infusion. The black arrows refer to degraded elastic fibers. Low-magnification images in O show the entire vascular wall at the site of analysis. Scale bar of high-magnification images in O, 100μm and 25μm. (P) Grade of elastin degradation in the aortic wall. Data are presented as mean ± SEM. Unpaired two-tailed Student's t tests were used in (E-J). Two-way ANOVA followed by Bonferroni post hoc analysis was applied in (K). Unpaired two-tailed Student's t test with Welch's correction was used in (P). A.U. indicates arbitrary unit.

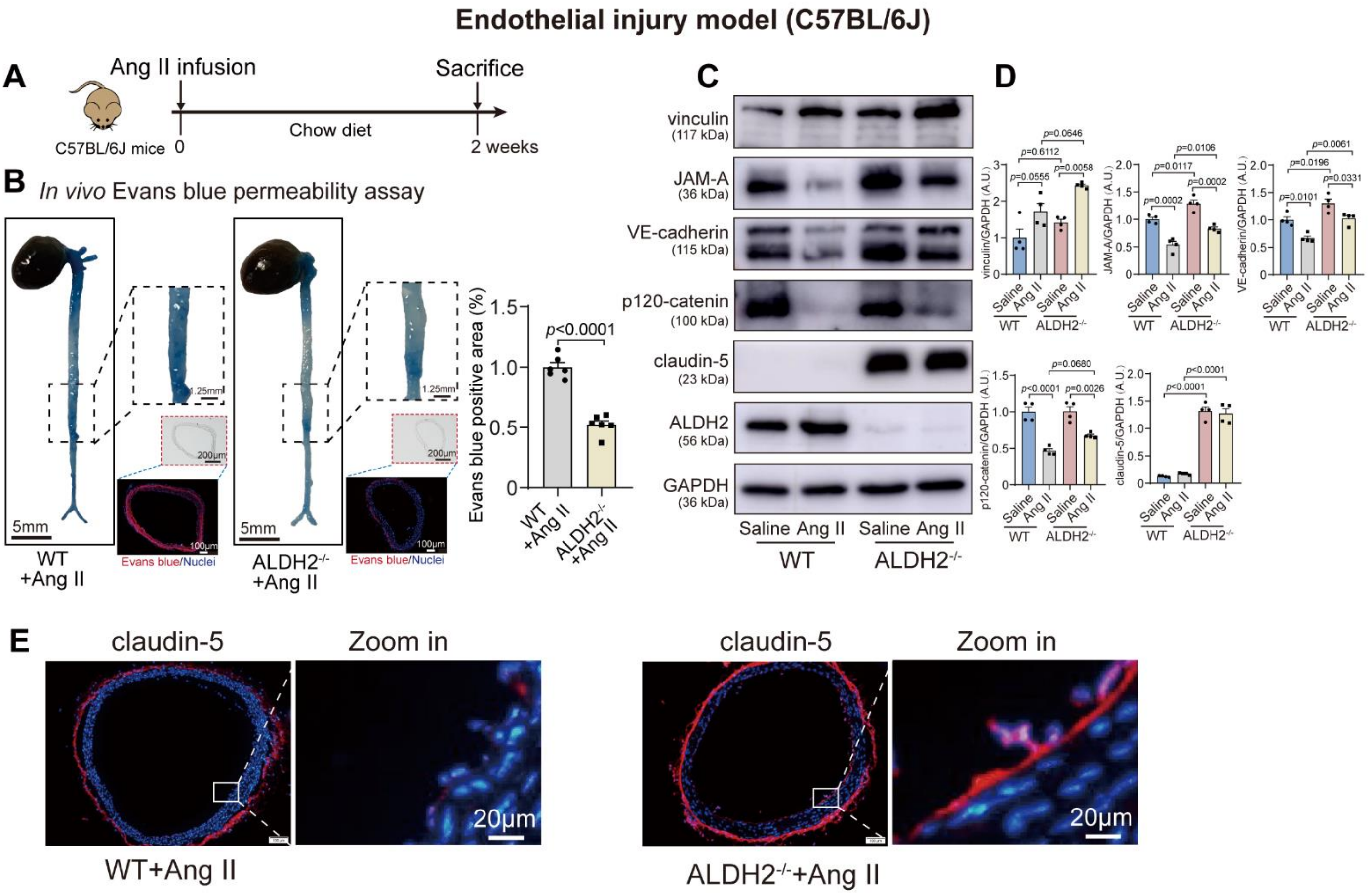

Figure S6: ALDH2 knockout alleviates Ang II–induced endothelial barrier dysfunction *in vivo*. (A) Schematic protocol exhibiting infusion of Ang II or saline in C57BL/6J mice for 2 weeks. (B) Permeability of aortic intimal barrier (to Evans blue dye) and quantification the Evans blue dye in WT and ALDH2$^{-/-}$ mice, and the autofluorescence of the dye in aortic cross-section images. WT+ Ang II, n=6; and ALDH2$^{-/-}$ + Ang II, n=6. Scale bar, 5mm. (C-D) Representative immunoblots and quantification for vinculin, JAM-A, VE-cadherin, p120-cadherin, claudin-5 and ALDH2 in the indicated groups (n=3 per group). (E) Representative images of claudin-5 (red) in WT and ALDH2$^{-/-}$ mice 2 weeks post-Ang II infusion. Scale bar of low-magnification images, 100μm. Scale bar of zoom in images, 20μm. Data are presented as mean ± SEM. Unpaired two-tailed Student's t tests were used in (B). Two-way ANOVA followed by Bonferroni post hoc analysis was applied in (D). A.U. indicates arbitrary unit.

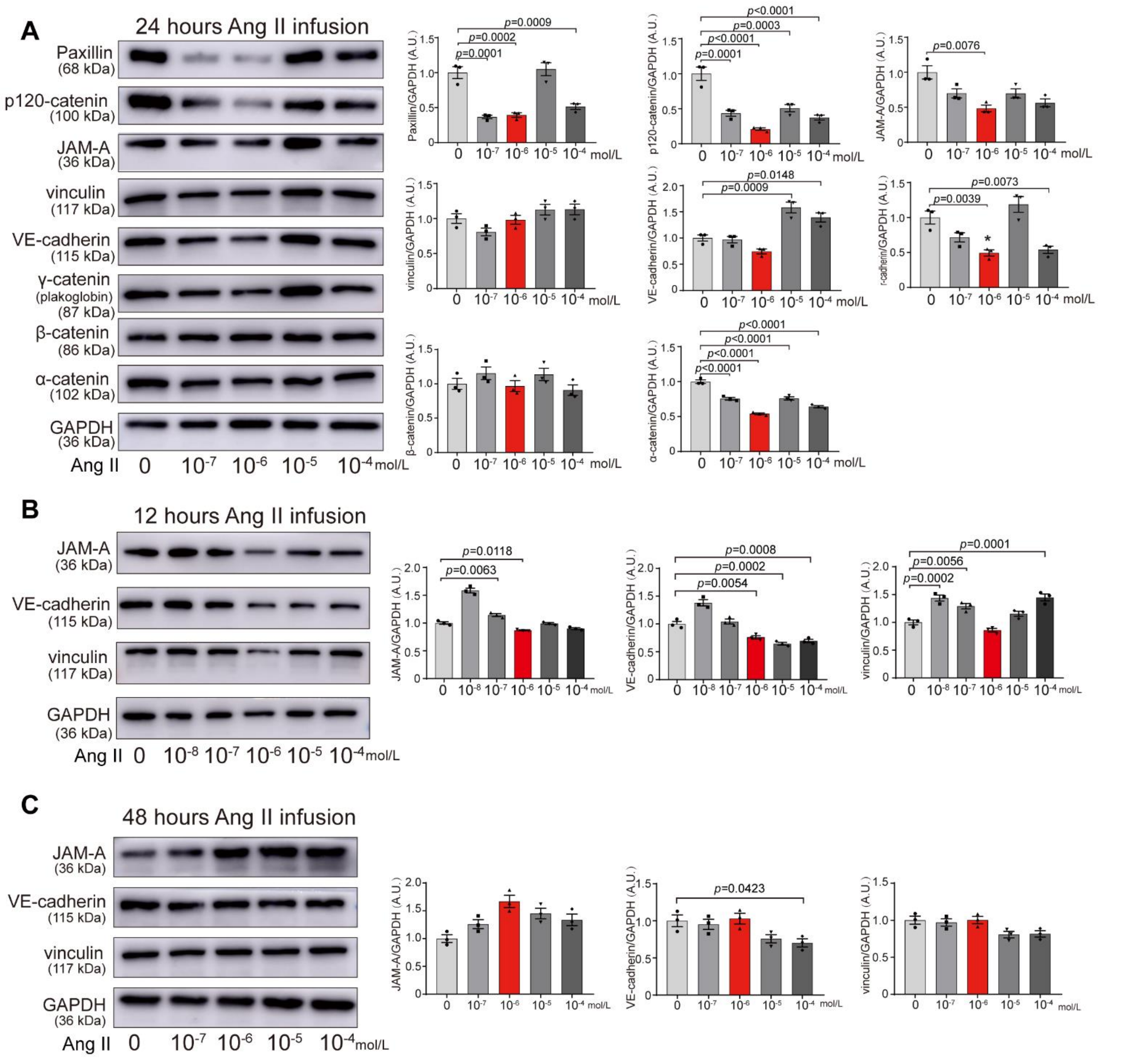

Figure S7: Optimal doses and time lengths of Ang II treatment in endothelial cells as *in vitro* model. (A) Representative immunoblots and quantifications for Paxillin, p120-catenin, JAM-A, vinculin, VE-cadherin, γ-catenin, β-catenin and α-catenin in HUVECs subjected to different concentrations gradient of Ang II treatment for 24 hours (n=3 per group). (B) Representative immunoblots and quantifications for JAM-A, VE-cadherin and vinculin in HUVECs subjected to different concentration gradient of Ang II treatment for 12 hours (n=3 per group). (C) Representative immunoblots and quantifications for JAM-A, VE-cadherin and vinculin in HUVECs subjected to different concentration gradient of Ang II treatment for 48 hours (n=3 per group). Data are presented as mean ± SEM. Kruskal-Wallis test followed by Bonferroni post hoc test was applied in JAM-A of (A). One-way ANOVA followed by Bonferroni post hoc tests were applied in (B) (C) and the rest markers of (A). A.U. indicates arbitrary unit.

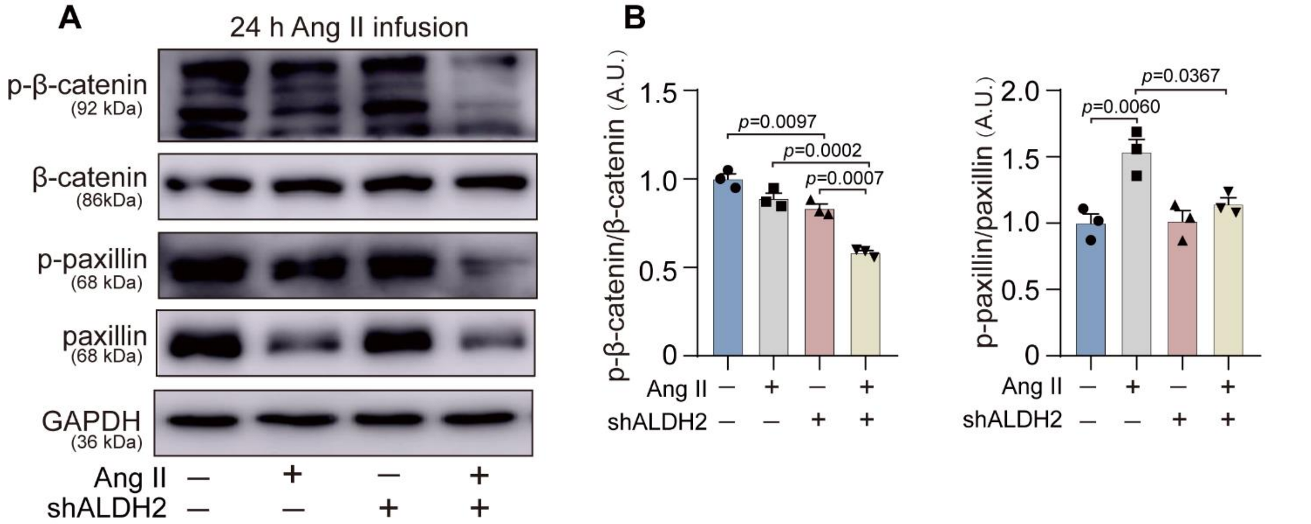

Figure S8: Expression of other ECs adhesion molecules *in vitro*. (A-B) Representative immunoblots and quantification for p-β-catenin, β-catenin, p-paxillin and paxillin in the indicated groups for 24 hours (n=3 per group). Data are presented as mean ± SEM. Two-way ANOVA followed by Bonferroni post hoc analysis was applied in (B). A.U. indicates arbitrary unit.

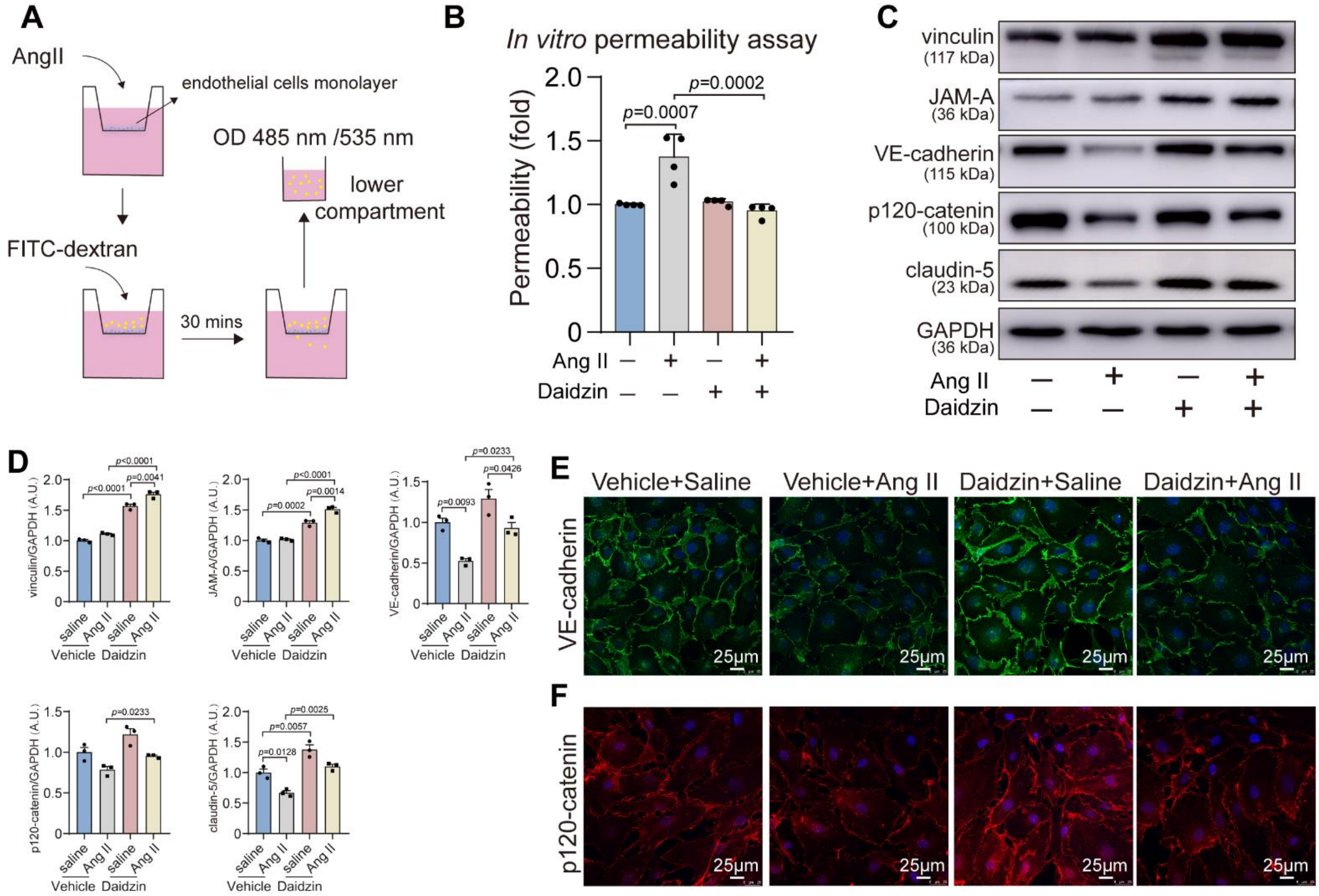

Figure S9: ALDH2 inhibition alleviates Ang II–induced endothelial barrier dysfunction *in vitro*. (A)

Experimental protocol: HUVECs were allowed to form a monolayer and were stimulated with Ang II (10^{-6} M) for 24 hours. The amount of FITC-dextran in the bottom chamber was determined by fluorometric analysis. (B) *In vitro* permeability assay for HUVECs monolayers [Daidzin or DMSO (Vehicle)] after treatment with Ang II (10^{-6} M) for 24 hours (n=4 per group). (C-D) Representative immunoblots and quantification for vinculin, JAM-A, VE-cadherin, p120-catenin and claudin-5 in the indicated groups (n=3 per group). (E) VE-cadherin-specific antibody staining (green, scale bars, 25μm) and (F) p120-catenin-specific antibody staining (red, scale bars, 25μm) of Ang II–transfected HUVECs. Data are presented as mean ± SEM. Two-way ANOVA followed by Bonferroni post hoc analyses were applied in (B) and (D). A.U. indicates arbitrary unit.

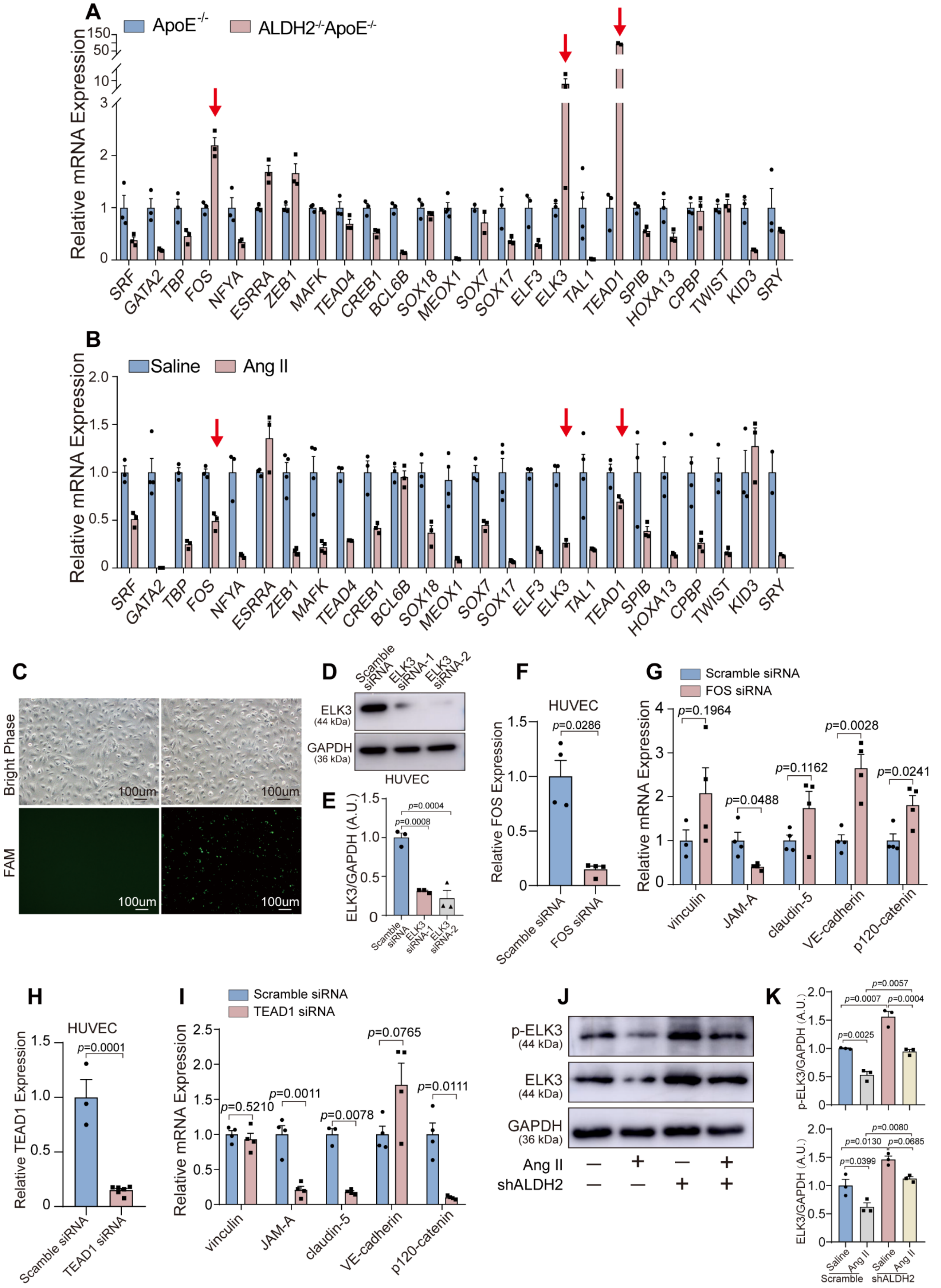
A
ApoE-/-
ALDH2-/-ApoE-/-
Relative mRNA Expression
SRF GATA2 TBP FOS NFYA ESRRA ZEB1 MAFK TEAD4 CREB1 BCL6B SOX18 MEOX1 SOX7 SOX17 ELF3 ELK3 TAL1 TEAD1 SPIB HOXA13 CPBP TWIST KID3 SRY
B
Saline
Ang II
Relative mRNA Expression
SRF GATA2 TBP FOS NFYA ESRRA ZEB1 MAFK TEAD4 CREB1 BCL6B SOX18 MEOX1 SOX7 SOX17 ELF3 ELK3 TAL1 TEAD1 SPIB HOXA13 CPBP TWIST KID3 SRY
C
Bright Phase
FAM
100um
D
Scamble siRNA
ELK3 siRNA-1
ELK3 siRNA-2
ELK3 (44 kDa)
GAPDH (36 kDa)
HUVEC
E
ELK3/GAPDH (A.U.)
p=0.0004
p=0.0008
F
HUVEC
Relative FOS Expression
p=0.0286
Scamble siRNA
FOS siRNA
G
Scramble siRNA
FOS siRNA
Relative mRNA Expression
p=0.1964
p=0.0488
p=0.1162
p=0.0028
p=0.0241
vinculin
JAM-A
claudin-5
VE-cadherin
p120-catenin
H
HUVEC
Relative TEAD1 Expression
p=0.0001
Scamble siRNA
TEAD1 siRNA
I
Scramble siRNA
TEAD1 siRNA
Relative mRNA Expression
p=0.5210
p=0.0011
p=0.0078
p=0.0765
p=0.0111
vinculin
JAM-A
claudin-5
VE-cadherin
p120-catenin
J
p-ELK3 (44 kDa)
ELK3 (44 kDa)
GAPDH (36 kDa)
Ang II
shALDH2
K
p-ELK3/GAPDH (A.U.)
p=0.0057
p=0.0007
p=0.0004
p=0.0025
ELK3/GAPDH (A.U.)
p=0.0080
p=0.0130
p=0.0685
p=0.0399
Saline
Ang II
Scramble
shALDH2

Figure S10: (A) RT-qPCR of the predicted 25 transcription factors on aortas from $ApoE^{-/-}$ and $ALDH2^{-/-}ApoE^{-/-}$ mice (n=3 per group). (B) RT-qPCR of the predicted 25 transcription factors on Ang II-treated HUVECs (n=3 per group). (C) Representative bright phase and fluorescence images of HUVECs transfected with fluorescein amidite (FAM). Scale bars, 100µm. (D-E) Representative immunoblots and quantification for ELK3 in HUVECs transfected with negative control siRNA (scramble siRNA) or ELK3 siRNA (n=3 per group). (F) RT-qPCR of FOS in HUVECs transfected with scramble siRNA or FOS siRNA (n=4 per group). (G) RT-qPCR of vinculin, JAM-A, claudin-5, VE-cadherin and p120-catenin in HUVECs transfected with scramble siRNA or FOS siRNA (n=4 per group). (H) RT-qPCR of TEAD1 in HUVECs transfected with scramble siRNA or TEAD1 siRNA (n=4 per group). (I) RT-qPCR of vinculin, JAM-A, claudin-5, VE-cadherin and p120-catenin in HUVECs transfected with scramble siRNA or TEAD1 siRNA (n=4 per group). (J-K) Representative immunoblots and quantification for p-ELK3 and ELK3 in HUVECs transfected with negative control shRNA (scramble) or ALDH2 shRNA after 24 hours of Ang II (10^{-6} M) treatment (n=3 per group). Data are presented as mean ± SEM. One-way ANOVA followed by Bonferroni post hoc test was applied in (E). Mann-Whitney U test was applied in (F). Unpaired two-tailed Student's t tests with Welch's correction were used in JAM-A of (G) and claudin-5, p120-catenin of (I). Unpaired two-tailed Student's t tests were applied in (H), the rest markers of (G) and (I). Two-way ANOVA followed by Bonferroni post hoc analyses were applied in (K). A.U. indicates arbitrary unit.

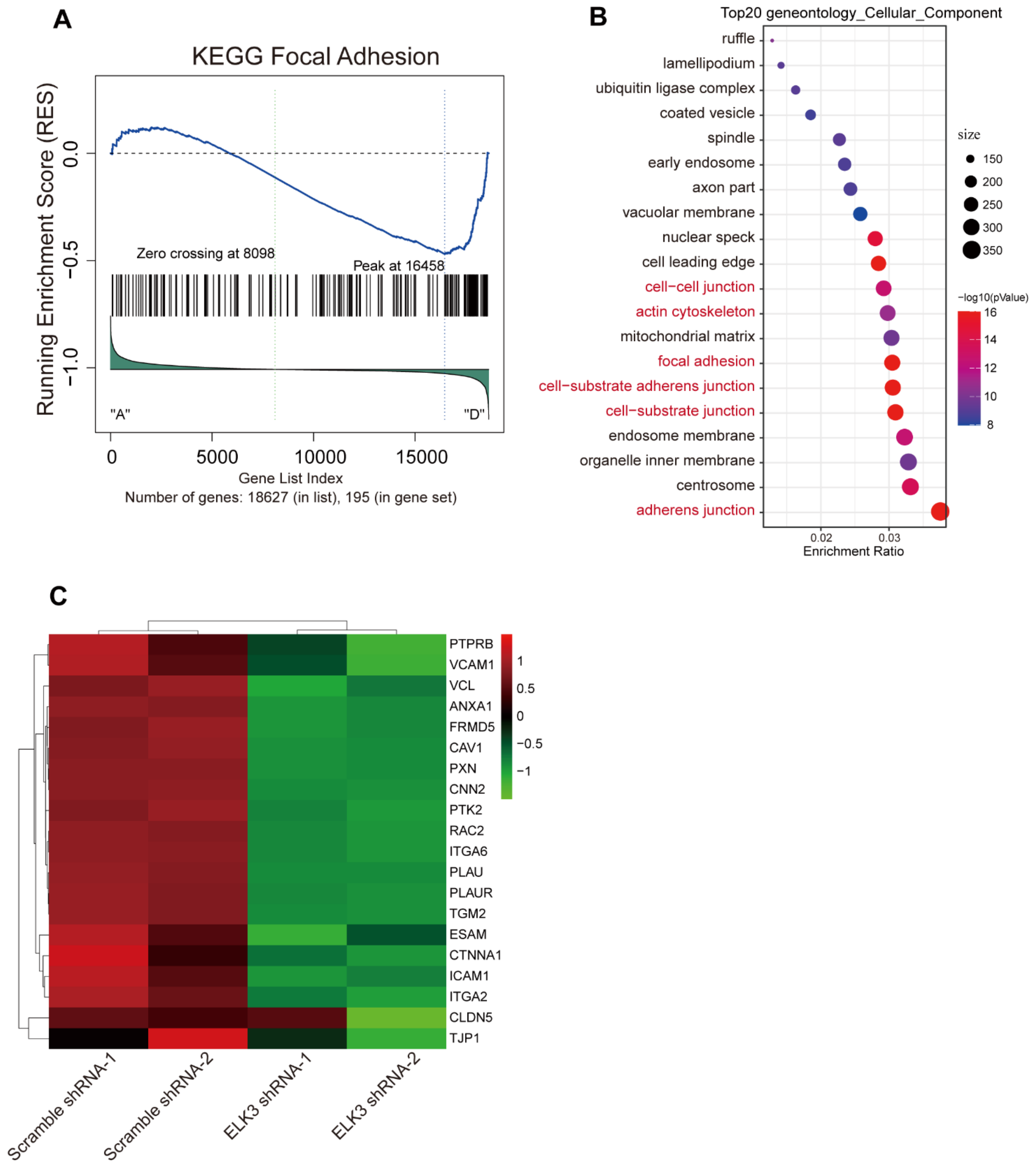

Figure S11: ELK3 preserved endothelial barrier function in the MDA-MB-231 cell line. (A) Gene set enrichment analysis (GSEA) was performed on the ELK3 knockdown regulated expression profile using the GSE83325 data set. (B) Top 20 enriched downregulated gene ontology (GO) terms related to cellular component in ELK3 knockdown target genes. (C) Heat map showing the genes related to endothelial barrier regulated by ELK3 knockdown.

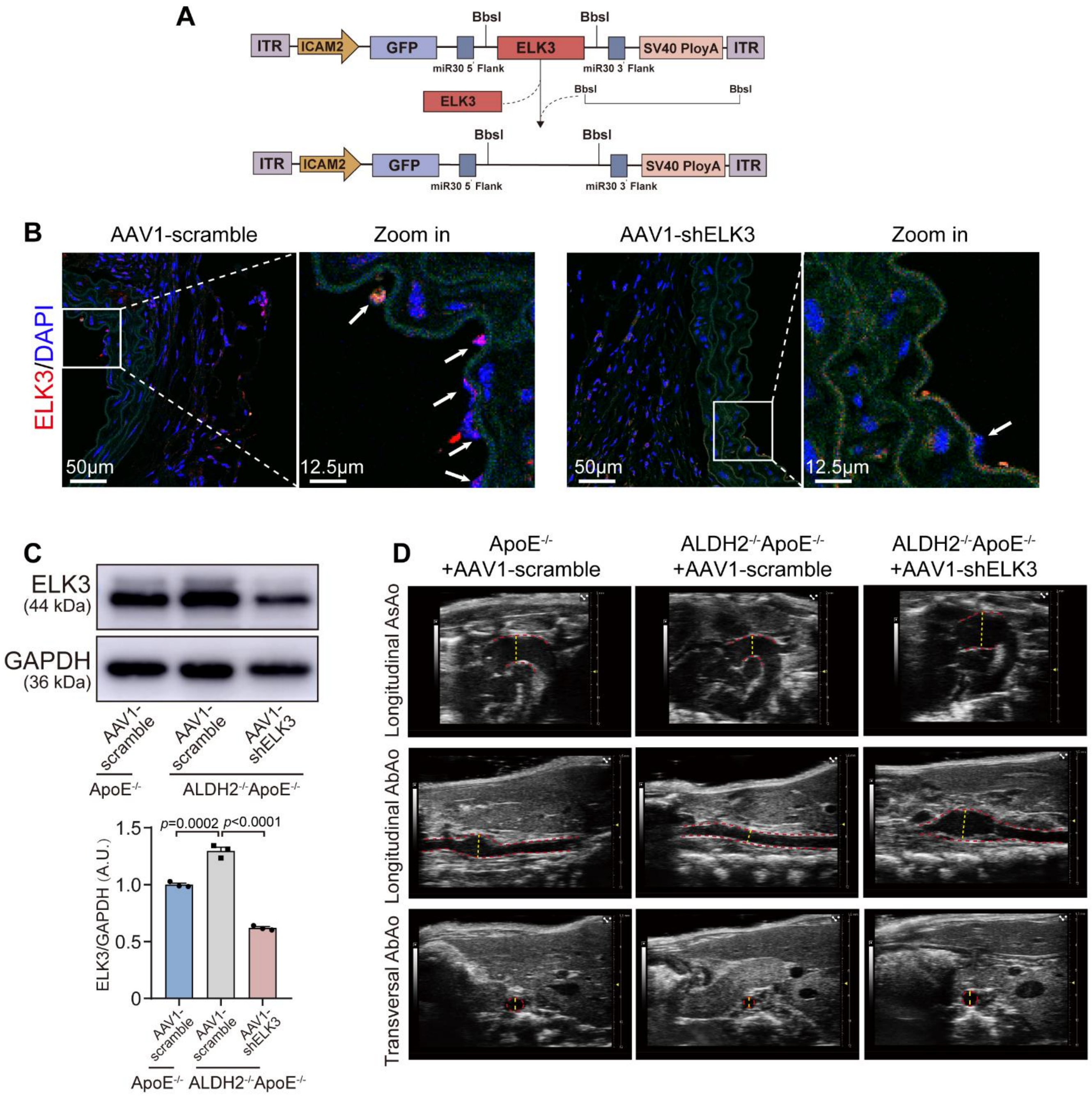

Figure S12: (A) Vector construction of AAV1-shELK3. (B) Representative images of immunofluorescence staining of ELK3 in the aortic intima following tail vein injection of ICAM2 promoter-driven AAV1 carrying ELK3-shRNA or scramble-shRNA. Scale bar, 50μm and 12.5μm. (C) ELK3 expression in the aortic intima by western blot in 3 animal groups following tail vein injection of ICAM2 promoter-driven AAV1 carrying ELK3-shRNA or scramble-shRNA. (D) Representative ascending aortic (AsAo) and abdominal aorta (AbAo) ultrasound images in 3 animal groups at 4 weeks following Ang II infusion. Data are presented as mean ± SEM. One-way ANOVA followed by Bonferroni post hoc test was applied in (C). A.U. indicates arbitrary unit.

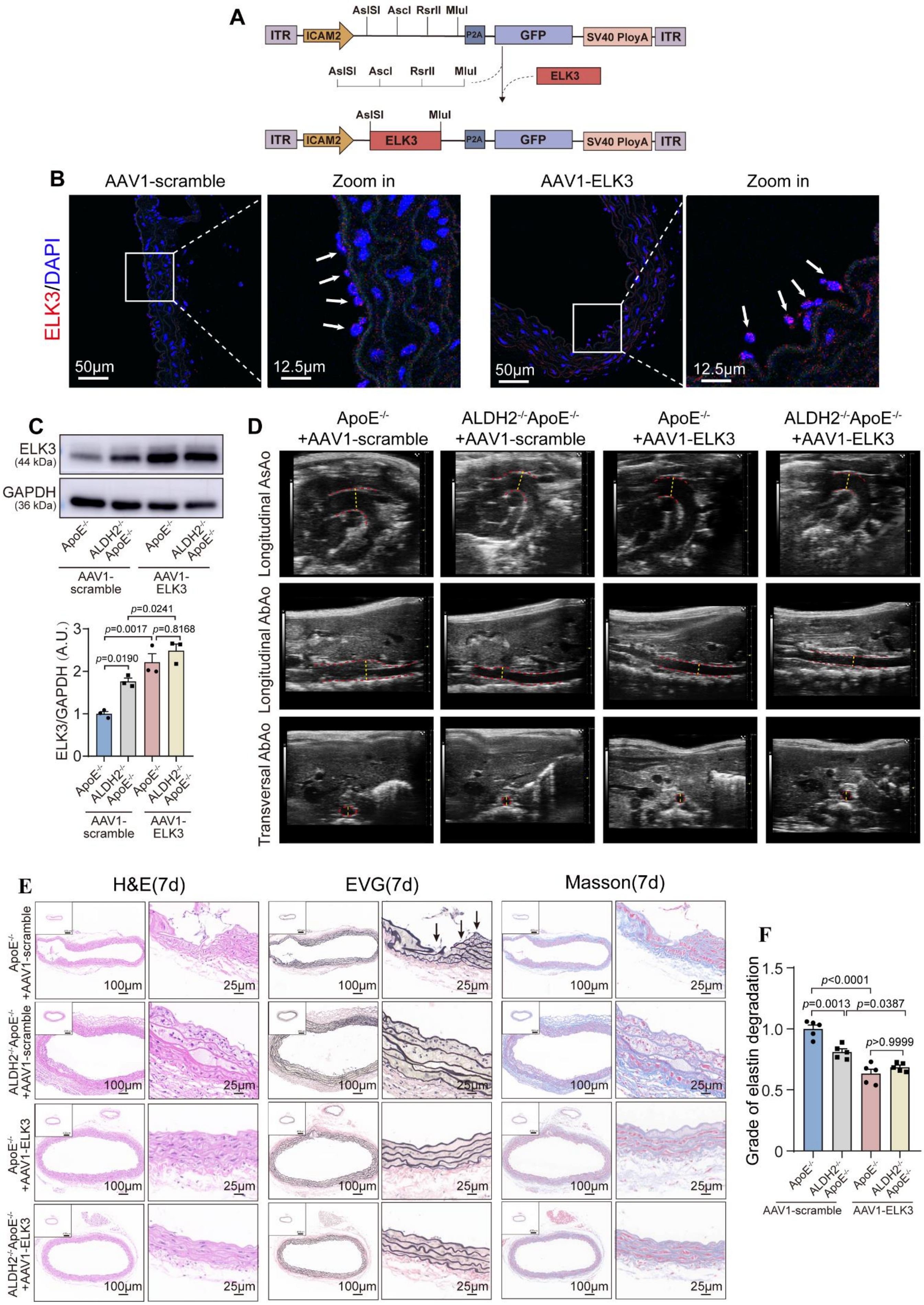
A
ITR
ICAM2
AsISI
AscI
RsrII
MluI
P2A
GFP
SV40 PloyA
ITR
ELK3
B
AAV1-scramble
Zoom in
AAV1-ELK3
Zoom in
ELK3/DAPI
50μm
12.5μm
C
ELK3 (44 kDa)
GAPDH (36 kDa)
ApoE-/-
ALDH2-/- ApoE-/-
AAV1-scramble
AAV1-ELK3
ELK3/GAPDH (A.U.)
p=0.0241
p=0.0017
p=0.8168
p=0.0190
D
ApoE-/- +AAV1-scramble
ALDH2-/-ApoE-/- +AAV1-scramble
ApoE-/- +AAV1-ELK3
ALDH2-/-ApoE-/- +AAV1-ELK3
Longitudinal AsAo
Longitudinal AbAo
Transversal AbAo
E
H&E(7d)
EVG(7d)
Masson(7d)
ApoE-/- +AAV1-scramble
ALDH2-/-ApoE-/- +AAV1-scramble
ApoE-/- +AAV1-ELK3
ALDH2-/-ApoE-/- +AAV1-ELK3
100μm
25μm
F
Grade of elastin degradation
p<0.0001
p=0.0013
p=0.0387
p>0.9999
AAV1-scramble
AAV1-ELK3

Figure S13: (A) Vector construction of AAV1-ELK3 overexpression plasmid. (B) Representative images of immunofluorescence staining of ELK3 in the aortic intima following tail vein injection of ICAM2 promoter-driven AAV1 carrying ELK3 or scramble. Scale bar, 50μm and 12.5μm. (C) ELK3 expression in the aortic intima by western blot in 4 animal groups following tail vein injection of ICAM2 promoter-driven AAV1 carrying ELK3-shRNA or scramble-shRNA. (D) Representative ascending aortic (AsAo) and abdominal aorta (AbAo) ultrasound images in 4 animal groups after 7 days Ang II infusion. (E) Representative H&E, EVG staining and Masson staining of mouse abdominal aorta after 7 days post-Ang II treatment. The black arrows refer to degraded elastic fibers. Low-magnification images show the entire vascular wall at the site of analysis. Scale bar of high-magnification images in E, 100μm and 25μm. (F) Grade of elastin degradation in the aortic wall. Data are presented as mean ± SEM. Two-way ANOVA followed by Bonferroni post hoc analyses were applied in (C) (F). A.U. indicates arbitrary unit.

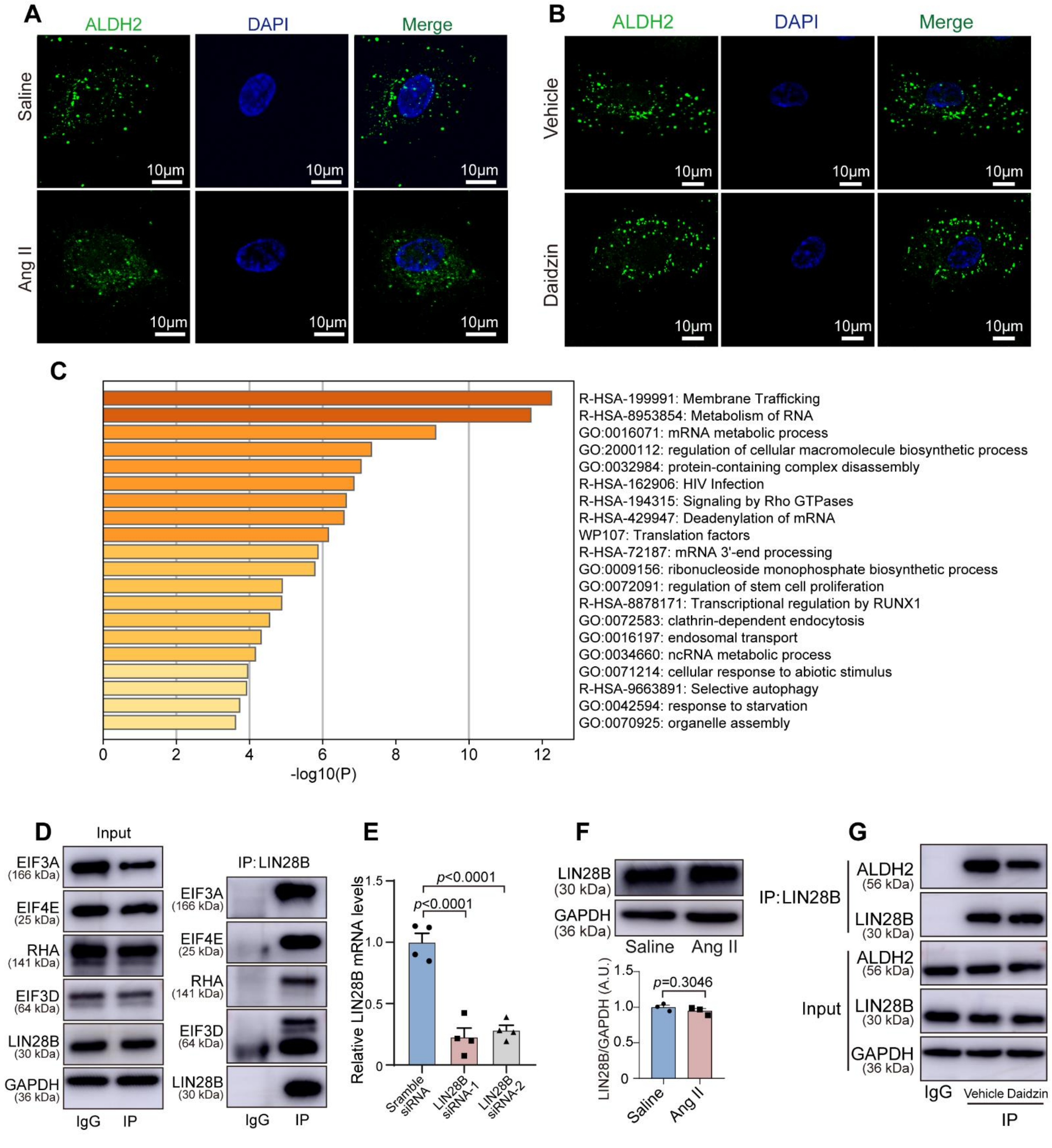
A
ALDH2
DAPI
Merge
Saline
Ang II
10μm
B
ALDH2
DAPI
Merge
Vehicle
Daidzin
10μm
C
R-HSA-199991: Membrane Trafficking
R-HSA-8953854: Metabolism of RNA
GO:0016071: mRNA metabolic process
GO:2000112: regulation of cellular macromolecule biosynthetic process
GO:0032984: protein-containing complex disassembly
R-HSA-162906: HIV Infection
R-HSA-194315: Signaling by Rho GTPases
R-HSA-429947: Deadenylation of mRNA
WP107: Translation factors
R-HSA-72187: mRNA 3'-end processing
GO:0009156: ribonucleoside monophosphate biosynthetic process
GO:0072091: regulation of stem cell proliferation
R-HSA-8878171: Transcriptional regulation by RUNX1
GO:0072583: clathrin-dependent endocytosis
GO:0016197: endosomal transport
GO:0034660: ncRNA metabolic process
GO:0071214: cellular response to abiotic stimulus
R-HSA-9663891: Selective autophagy
GO:0042594: response to starvation
GO:0070925: organelle assembly
-log10(P)
D
Input
IP: LIN28B
EIF3A (166 kDa)
EIF4E (25 kDa)
RHA (141 kDa)
EIF3D (64 kDa)
LIN28B (30 kDa)
GAPDH (36 kDa)
IgG
IP
E
Relative LIN28B mRNA levels
p<0.0001
p<0.0001
Sramble siRNA
LIN28B siRNA-1
LIN28B siRNA-2
F
LIN28B (30 kDa)
GAPDH (36 kDa)
Saline
Ang II
LIN28B/GAPDH (A.U.)
p=0.3046
G
IP: LIN28B
Input
ALDH2 (56 kDa)
LIN28B (30 kDa)
GAPDH (36 kDa)
IgG
Vehicle
Daidzin
IP

Figure S14: (A) Immunofluorescence staining for ALDH2 in HUVECs receiving saline and Ang II treatment. Scale bars, 10μm. (B) Immunofluorescence staining for ALDH2 in HUVECs receiving vehicle and Daidzin treatment. Scale bars, 10μm. (C) A list of KEGG enrichment analysis pathways in HUVECs identified in the protein mixture enriched by LIN28B antibody. (D) Co-immunoprecipitation assay of LIN28B and EIF3A、EIF4E、RHA、EIF3D in HUVECs cell line (n=3 per group). (E) RT–qPCR of LIN28B in HUVECs cell line treated with LIN28B siRNA (n=4 per group). (F) Representative immunoblots and quantification for LIN28B in HUVECs cell line treated with Ang II (n=3 per group). (G) Co-immunoprecipitation assay of ALDH2 and LIN28B in HUVECs cell line treated with vehicle and Daidzin (n=3 per group). Data are presented as mean ± SEM. One-way ANOVA followed by Bonferroni post hoc test was applied in (E). Unpaired two-tailed Student's t test was used in (F). A.U. indicates arbitrary unit.

Table S1: Patient information for human aortic samples (n=12).

AD1	AAD2	AAD3	AAD4	AAD5	AAD6	Control1	Control2	Control3	Control4
ale	Female	Female	Male	Male	Female	Male	Male	Male	Male
llow	Yellow	Yellow	Yellow	Yellow	Yellow	Yellow	Yellow	Yellow	Yellow
47	54	48	60	59	48	48	47	45	75
rtic ection ford B)	aortic dissection (Stanford B)	aortic dissection (Stanford A)	aortic dissection (Stanford A)	aortic dissection (Stanford A)	aortic dissection (Stanford A)	organ donors	organ donors	organ donors	organ donors
o	No	No	No	No	No	Yes	Yes	No	No
es	No	Yes	No	No	No	Yes	Yes	No	No
es	Yes	No	Yes	Yes	Yes	No	Yes	Yes	No
es	No	No	No	No	No	No	No	No	Yes
o	No	No	No	No	No	No	No	No	No
o	No	No	No	No	No	No	No	No	No

Table S2: Sequences of siRNA/shRNA for gene knockdown.

	Sequence (5' → 3')
ELK3-homo-228 siRNA	sense sequence: GGCAGAAGUUUGUGUACAATT<br>antisense strand: UUGUACACAAACUUCUGCCTT
ELK3-homo-40 siRNA	sense sequence: GCUGCUGGAUCAGAAACAUTT<br>antisense strand: AUGUUUCUGAUCCAGCAGCTT
Elk3-Mus-662 siRNA	sense sequence: GCCAAGAUCUCCUCUUUAATT<br>antisense strand: UUAAAGAGGAGAUCUUGGCTT
Elk3-Mus-228 siRNA	sense sequence: GGCAGAAGUUUGUGUACAATT<br>antisense strand: UUGUACACAAACUUCUGCCTT
Elk3-Mus-537 siRNA	sense sequence: GGACUGUGAUCAGGUUUGUTT<br>antisense strand: ACAAACCUGAUCACAGUCCTT
TEAD1-homo-1015-siRNA	sense sequence: GCCACUGCCAUUCAUAACATT<br>antisense strand: UGUUAUGAAUGGCAGUGGCTT
TEAD1-homo-1492-siRNA	sense sequence: GCUGAUUUAAACUGCAAUATT<br>antisense strand: UAUUGCAGUUUAAAUCAGCTT
TEAD1-Homo-1019-siRNA	sense sequence: CUGCCAUUCAUAACAAGCUTT<br>antisense strand: AGCUUGUUAUGAAUGGCAGTT
FOS-homo-274-siRNA	sense sequence: GGGAUAGCCUCUCUUACUATT<br>antisense strand: UAGUAAGAGAGGCUAUCCCTT

FOS-homo-707-siRNA	sense sequence: GACAGACCAACUAGAAGAUTT<br>antisense strand: AUCUUCUAGUUGGUCUGUCTT
FOS-homo-584-siRNA	sense sequence: CAAGGUGGAACAGUUAUCUTT<br>antisense strand: AGAUAACUGUUCCACCUUGTT
LIN28B-homo-siRNA-1	GGATTCATCTCCATGATAA
LIN28B-homo-siRNA-2	CTTGGATATTCCAGTCGAT
ALDH2-homo-463 siRNA	sense sequence: GUGCCUCAGAAGAACUCAUTT<br>antisense strand: AUGAGUUCUUCUGAGGCACTT
	Sequence (5' → 3')
ALDH2 shRNA	ACTGAAATGTCTCCGCTATTACTAGTGAAGCCACA<br>GATGTAGTAATAGCGGAGACATTTCAGG
ELK3 shRNA	CCAGGACTGTGATCAGGTTTGTTAGTGAAGCCACA<br>GATGTAACAAACCTGATCACAGTCCTGA

Table S3: Sequence of primers used in RT-qPCR analysis.

The sequence of primers used in qPCR analysis	
	Sequence (5' → 3')
Mus-Spib-148F	GGGGCCTTGACTCTACATGG
Mus-Spib-148R	TAGGTGGAGGGGTTAGGACC
Mus-Hoxa13-160F	GTCTCCCATCCTTCAGACGC
Mus-Hoxa13-160R	CAGAGAGGTTTGTCGTGGCT
Mus-Twist1-110 F	CAGCGGGTCATGGCTAACG
Mus-Twist1-110 R	TTGCTCAGCTTGTCCGAGG
Mus-CPBP(KLF6)-196F	CAGGAAGACCTGTGGACCAA
Mus-CPBP(KLF6)-196R	TGGTCGTGGGTGAAAGTTCC
Mus-KID3(ZNF354C)-72F	CAGAGTCACCCAAGGAAACCA
Mus-KID3(ZNF354C)-72R	TGAACAAGGGTTTCTCCGGC
Mus-Sry-119F	CATCGGAGGGCTAAAGTGTCA
Mus-Sry-119R	TCTTGCCTGTATGTGATGGCA
Mus-Gata2-151F	CCGACGAGGTGGATGTCTTC
Mus-Gata2-151R	CTGTGCAACAAGTGTGGTCG
Mus-c-fos-194 F	AGAGCGGGAATGGTGAAGAC
Mus-c-fos-194 R	AGTTGATCTGTCTCCGCTTGG
Mus-Srf-102F	CGGGTACCACCTCCACAATC
Mus-Srf-102R	ACACTGGTGCCAGGTAGTTG
Mus-Tbp-79F	CAGACCCCACAACTCTTCCAT
Mus-Tbp-79R	TTGGTGAAGGGTACAAGGGG
Mus-Nfya-119F	ACATCAACAGGCCAACCCAT
Mus-Nfya-119R	CCAGGAGGCACCAACTGTAT
Mus-Esrra-157F	AGTGTGAGATCACCAAGCGG
Mus-Esrra-157R	AAAGGCAAAGGGTCCACCTC
Mus-Zeb1-141F	GGGACCTCAATGCACTTCCA
Mus-Zeb1-141R	GTGGCTGACTGGGAGACAAA
Mus-Mafk-138F	ACAGAAAGAGGAGCTGGAACG
Mus-Mafk-138R	GTGCGAGCGAAGGTCTGTA
Mus-Tead4-160F	TCGACAATGATGCAGAGGGT

Mus-Tead4-160R	CTGAGCTTGATATGGCGTGC
Mus-Creb1-79F	TGGAGTTGTTATGGCGTCCTC
Mus-Creb1-79R	TAGACGGACCTCTCTCTTCCG
Mus-Bcl6b-169F	TCACCGTTTCATCCAAGCCA
Mus-Bcl6b-169R	ACTTGGGGAACCTTGACTGC
Mus-Sox18-96F	CGGATCTGCACAACGCAGTA
Mus-Sox18-96R	GTTCGGCCTCTTCCACGAA
Mus-Meox1-171F	ACTCAGAAGATAGCAGCGCC
Mus-Meox1-171R	GCTGCTCGTTGAAGATTCGC
Mus-Sox7-95F	TCACCTCCCCCATCTACCAG
Mus-Sox7-95R	GGCCAAGGGCTAAAGAACCT
Mus-Sox17-104F	GTCTTGGAAGGCGTTGACCT
Mus-Sox17-104R	GCCGGTACTTGTAGTTGGGG
Mus-Elf3-88F	CATCCTAATCCACCCCGAGC
Mus-Elf3-88R	GGCCTCTGAGCGAAGAAACT
Mus-Elk3-88 F	CTGGAGCAGCCTTAGTCCTG
Mus-Elk3-88 R	GTTGAGCAGTGTGGGGAACT
Mus-Tal1-81F	CACCTAGTCCTGCTCAACGG
Mus-Tal1-81R	CGCTCCTAGCTCGATGACG
Mus-Tead1-174F	TCTGGGCGGACTTAAACTGC
Mus-Tead1-174R	GACCATTCTCGAACCTCGCA
Mus-p120-catenin (Ctnnd1)-183F	GGAAGCTCAAGGGTATCCCG
Mus-p120-catenin (Ctnnd1)-183R	TATCACGAGCCTTTCGGAGC
Mus-paxillin (Pxn)-174F	ACTTGACCGGCTGTTACTGG
Mus-paxillin (Pxn)-174R	GGCTTCTCTTTCACCAGGGG
Mus-VE-cadherin (Cdh5)-103F	AAGACATCCGAGTGGGCAAG
Mus-VE-cadherin (Cdh5)-103R	CTGTACTCGCCCTGCATGAT
Mus-vinculin (Vcl)-184F	GACTTGCGAAGACAGGGGAA
Mus-vinculin (Vcl)-184R	TGGGGTTATCAATCCACCGC
Mus-JAMA(F11r)-92 F	TACAAGGCAAGGGTTCGGTG
Mus-JAMA(F11r)-92R	GAAGCCAGAGTAGGTGCAGG
Mus-claudin5(Cldn5)-137F	GTTAAGGCACGGGTAGCACT
Mus-claudin5(Cldn5)-137R	TACTTCTGTGACACCGGCAC
Homo-SRF-141F	CCTCAACTCGCCAGACTCTC
Homo-SRF-141R	GGCTTCAGTGTGTCCTTGGT
Homo-GATA2-113F	CAGGGCAACCCCTACTATGC
Homo-GATA2-113R	CTGTGCAACAAGTGTGGGC
Homo-TBP-80F	TAAGAGAGCCACGAACCACG
Homo-TBP-80R	GGACTGTTCTTCACTCTTGGC
Homo-FOS-105F	TACTACCACTCACCCGCAGA
Homo-FOS-105R	CGTGGGAATGAAGTTGGCAC
Homo-NFYA-73F	TGGTCATGATGGTTCCTGGG
Homo-NFYA-73R	AGCATCTCTGCTCCAGGTAGA
Homo-ESRRA-72F	CACTATGGTGTGGCATCCTGT
Homo-ESRRA-72R	GTACTCGATGCTCCCCTGGA
Homo-ZEB1-126F	AATTCACAGTGGAGAGAAGCCA
Homo-ZEB1-126R	GGTCGCCCATTCACAGGTAT

Homo-MAFK-83F	AATCCCAAACCGAATAAGGCA
Homo-MAFK-83R	GACACCAGCTCATCATCGCT
Homo-TEAD4-162F	CTCCAGCCAGTATGAGAGCC
Homo-TEAD4-162R	ATGTACTCACAGAGCGGGGA
Homo-CREB1-73F	CCAGGAGTGCCAAGGATTGAA
Homo-CREB1-73R	CCGTTACAGTGGTGATGGCA
Homo--BCL6B-137F	AGCACACAAGGCAGTTCTCA
Homo--BCL6B-137R	TGAAGTCCAATAGAGGGGCG
Homo-SOX18-148F	CTGTACTACGGCACCCTGG
Homo-SOX18-148R	GGTACTGGTCGAACTCGGTG
Homo-MEOX1-70F	CCAGGCGATGACTACGGG
Homo-MEOX1-70R	TTCTCCGCCTGGATGATTTCT
Homo-SOX7-188F	CGAGCTGTCGGATGGACAAT
Homo-SOX7-188R	TCCACGACTTTCCCAGCATC
Homo-SOX17-100F	GGACCGCACGGAATTTGAAC
Homo-SOX17-100R	GCTGTCGGGGAGATTCACAC
Homo-ELF3-137F	GCAAGCGCCATTGACTTCTC
Homo-ELF3-137R	GAGCTGGAAGTGAGGTCTCG
Homo--ELK3-77F	AATGGATTGCTTCTGACTCCG
Homo--ELK3-77R	AGCGGAGCAACTGGACTAAG
Homo-TAL1-140F	AGTTGTGCGGCGTATCTTCA
Homo-TAL1-140R	CCAGGCGGAGGATCTCATTC
Homo-TEAD1-179F	CTCAGGACAGGCAAGACGAG
Homo-TEAD1-179R	TTATGAATGGCAGTGGCCGA
Homo-SPIB-117F	AGTTCTCCTCCAAGCACAAGG
Homo-SPIB-117R	CGGTCTTGGCGTAGTTTCGG
Homo-HOXA13-78F	ACTTGAACGGGAATACGCCA
Homo-HOXA13-78R	GAGAGATTCGTCGTGGCTGA
Homo-TWIST1-173F	GGACAGTGATTCCCAGACGG
Homo-TWIST1-173R	CCTTTCAGTGGCTGATTGGC
Homo-CPBP(KLF6)-97F	TCTCCCACGGCCAAGTTTAC
Homo-CPBP(KLF6)-97 R	GAGAAGATGGAGGCGTGGAG
Homo-KID3(ZNF354C)-194F	GTGTTCTTCAGCCAGGACGA
Homo-KID3(ZNF354C)-194 R	AGACGGGTATCTGAAGGGACT
Homo-SRY-82F	AGTGCAACTGGACAACAGGT
Homo-SRY-82R	TAAGTGGCCTAGCTGGTGCT
Homo-claudin5(CLDN5)-95F	GGCGTGCTCTACCTGTTTTG
Homo-claudin5(CLDN5)-95R	GGCACAGACGGGTCGTAAAA
Homo-JAM-A(F11R)-70F	AGCTGGTCTTTGATCCCCTG
Homo-JAM-A(F11R)-70 R	CCATACCCATTCCGTGCCTC
Homo-vinculin (VCL)-128F	TTGATGGGTCAAGGGGCATC
Homo-vinculin (VCL)-128R	CACCACCTCTGCCACTGTAA
Homo-VE-cadherin (CDH5)-232F	GCATCGGTTGTTCAATGCGT
Homo-VE-cadherin (CDH5)-232R	CGCTTCCACCACGATCTCAT
Homo-paxillin (PXN)-145F	TGTGGAGCCTTCTTTGGTCC
Homo-paxillin (PXN)-145R	GCGTGTTGAGGGCTGAGATA
Homo-p120-catenin (CTNND1)-81F	CAGATGATGGGACCACTCGG

Homo-p120-catenin (CTNND1)-81R	CTGGCTGTACTGTCCGTGTT
Homo-LIN28B-158F	GAAGGCGGGGCTAGCAA
Homo-LIN28B-158R	GGGCTTCCCTCTCGGTTTAT
